# *RNF115/BCA2* deficiency alleviated acute liver injury in mice by promoting autophagy and inhibiting inflammatory response

**DOI:** 10.1038/s41419-023-06379-7

**Published:** 2023-12-21

**Authors:** Jinqiu Feng, Shufang Ye, Bao Hai, Yaxin Lou, Mengyuan Duan, Pengli Guo, Ping Lv, Wenping Lu, Yingyu Chen

**Affiliations:** 1 Department of Immunology, Peking University School of Basic Medical Sciences; NHC Key Laboratory of Medical Immunology, Peking University, 38 Xueyuan Road, Beijing, 100191 China; 2 Department of Orthopedics, Peking University Third Hospital, 49 North Garden Road, Beijing, 100191 China; 3 Medical and Healthy Analytical Center, Peking University, 38 Xueyuan Road, Beijing, 100191 China; 4 Department of Hepatobiliary Surgery, First Medical Center, Chinese PLA General Hospital, 28 Fuxing Road, Beijing, 100853 China; 5 Center for Human Disease Genomics, Peking University, 38 Xueyuan Road, Beijing, 100191 China

**Keywords:** Experimental models of disease, Mechanisms of disease

## Abstract

The E3 ubiquitin ligase RING finger protein 115 (RNF115), also known as breast cancer-associated gene 2 (BCA2), has been linked with the growth of some cancers and immune regulation, which is negatively correlated with prognosis. Here, it is demonstrated that the *RNF115* deletion can protect mice from acute liver injury (ALI) induced by the treatment of lipopolysaccharide (LPS)/D-galactosamine (D-GalN), as evidenced by decreased levels of alanine aminotransaminase, aspartate transaminase, inflammatory cytokines (e.g., tumor necrosis factor α and interleukin-6), chemokines (e.g., MCP1/CCL2) and inflammatory cell (e.g., monocytes and neutrophils) infiltration. Moreover, it was found that the autophagy activity in *Rnf115*^−/−^ livers was increased, which resulted in the removal of damaged mitochondria and hepatocyte apoptosis. However, the administration of adeno-associated virus Rnf115 or autophagy inhibitor 3-MA impaired autophagy and aggravated liver injury in *Rnf115*^−/−^ mice with ALI. Further experiments proved that RNF115 interacts with LC3B, downregulates LC3B protein levels and cell autophagy. Additionally, *Rnf115* deletion inhibited M1 type macrophage activation via NF-κB and Jnk signaling pathways. Elimination of macrophages narrowed the difference in liver damage between *Rnf115*^*+/+*^ and *Rnf115*^−/−^ mice, indicating that macrophages were linked in the ALI induced by LPS/D-GalN. Collectively, for the first time, we have proved that *Rnf115* inactivation ameliorated LPS/D-GalN-induced ALI in mice by promoting autophagy and attenuating inflammatory responses. This study provides new evidence for the involvement of autophagy mechanisms in the protection against acute liver injury.

## Introduction

Acute liver injury (ALI) is a fatal illness marked by the abrupt development of jaundice, coagulopathy, and hepatic encephalopathy in individuals with no previous history of hepatic disease [1]. Drug-induced liver injury is the most common cause of liver failure. Hepatocyte injury or death leads to the release of damage-associated molecular patterns (DAMPs) [2]. Liver-resident Kupffer cells (macrophages) highly express various DAMP receptors (e.g., Toll-like receptor [TLR]-4, TLR9), thus mediating the immune response to injury. Subsequently, activated Kupffer cells secrete pro-inflammatory cytokines (e.g., tumor necrosis factor [TNF]-α and interleukin [IL]-6), reactive oxygen species (ROS), and chemokines (e.g., MCP1/CCL2) that amplify the pro-inflammatory signal and increase the recruitment of bone marrow-derived cells—mainly neutrophils and monocytes— to the liver, thereby enhancing the inflammatory impairment [3]. Co-administration of lipopolysaccharide (LPS)/D-galactosamine (D-GalN) is a well-established method to induce ALI in mice, which has been widely used to study the pathophysiological mechanisms of ALI and identify novel therapeutic strategies. D-GalN mediates the sensitivity of hepatocytes to LPS-induced cytotoxicity [4]. LPS binds to and activates macrophages that in turn produce pro-inflammatory cytokines (such as TNF-α), which induces hepatocyte apoptosis and liver injury. At the same time, activated macrophages secret chemokines (such as CCL2), which promotes the recruitment of monocytes and neutrophils, thereby amplifying liver injury [5–7], finally triggering severe acute liver failure in mice.

Cell autophagy is an evolutionarily conserved cellular degradation process induced under various cellular stress conditions including nutrient deficiency and infection [8]. Autophagy plays a critical role in cellular homeostasis through the degradation of protein aggregates, pathogens, lipids, and senescent/damaged subcellular organelles such as damaged mitochondria [9]. Autophagy dysfunction in the liver may lead to various liver diseases including non-alcoholic fatty liver disease, drug-induced liver injury, cholestasis, and hepatocellular carcinoma [10]. Mice treated with rapamycin or torin 1 (autophagy inducers) are protected against acetaminophen-induced liver injury, whereas treatment with chloroquine (autophagy inhibitor) exacerbates ALI in mice [11–13]. Autophagy defects in hepatocytes are associated with higher sensitivity to LPS/D-GalN injury and increased tissue damage, cell apoptosis, and death [14]. Therefore, targeting autophagy is a potential treatment strategy for ALI.

Upregulation of E3 ubiquitin ligase RNF115/BCA2—also known as Rab7-interacting RING finger protein (Rabring7), widely expressed in various tissues—is observed in different carcinomas, including breast cancer [15], lung cancer [16], and gastric cancer [17] and is negatively correlated with prognosis. It has been demonstrated that RNF115 catalyzes the ubiquitination of a series of proteins to modulate several signaling pathways [18] and thereby regulates multiple processes such as cell proliferation [19], tumorigenesis [16, 20], autophagy [17], and viral infections [21–23]. A recent study has demonstrated that RNF115 negatively regulates phagosome maturation and host response to bacterial infection [24]. It also inhibits the post-endoplasmic reticulum trafficking of TLRs and TLR-mediated immune responses by catalyzing the ubiquitination of the small GTPases RAB1A and RAB13 [25]. To date, the association of RNF115 with liver inflammatory diseases has not been reported.

In the present study, we generated *Rnf115* knockout (KO) mice to further investigate the pathophysiological functions of RNF115. To our knowledge, this is the first study to demonstrate that RNF115 negatively regulates LC3B expression and cell autophagy. Rnf115 deficiency exerts a protective effect in mice with LPS/D-GalN- induced ALI. *Rnf115*^*-/-*^ mice exhibited decreased sensitivity to LPS, accompanied by the inhibition of M1 macrophage activation. These findings provide a basis for the development of strategies to prevent or treat ALI by targeting RNF115.

## Results

### *Rnf115* deficiency alleviates lipopolysaccharide/D-galactosamine-induced acute liver injury in mice

*Rnf115* knockout mice were generated by CRISPR/Cas9-mediated genome editing. We designed two targets ending in NGG on both sides of exon 2 (59 bp), which caused a frameshift and led to the translational termination of RNF115 (amino acids 1–34; Fig. S1a). The *Rnf115* knockout was confirmed by performing PCR of mouse tail genomic DNA (Fig. S1b) and immunoblotting and quantitative RT-PCR analysis of bone marrow-derived macrophages (BMDMs; Fig. S1c and S1d). The resulting *Rnf115*^*-/-*^ mice did not exhibit spontaneous phenotypes compared with age-matched *Rnf115*^*+/+*^ littermate controls. Flow cytometry data indicated no significant difference in the proportion and number of T and B cells, macrophages, or neutrophils in different tissues between *Rnf115*^*+/+*^ and Rnf115^−/−^ mice (Fig. S2 and S3).

**Fig. 1:**
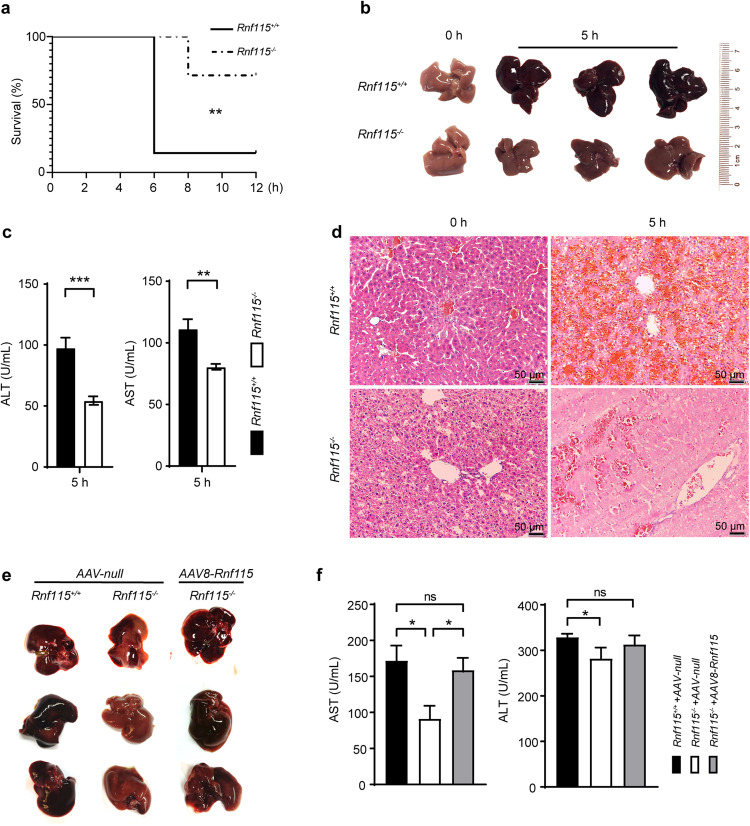
Genetic deletion of *Rnf115* alleviates acute liver injury caused by LPS/D-GalN in mice. **a** *Rnf115*^*+/+*^and *Rnf115*^*-/-*^ mice (*n* = 14) were intraperitoneally injected with LPS (30 μg/kg) and D-GalN (350 mg/kg) or phosphate-buffered saline (PBS), observed every 2 h, and the survival curve was outlined. **b** Representative livers from *Rnf115*^*+/+*^and *Rnf115*^*-/-*^ mice with or without LPS/D-GalN treatment for 5 h. **c** Serum ALT and AST levels from different groups. **d** Representative micrographs of livers stained with H&E from different groups. **e** Representative image of the livers from different groups. **f** Serum ALT and AST levels from mice in **e**. *p < 0.05, **p < 0.01, ***p < 0.001, ns: no significance.

Next, we investigated the effects of *Rnf115* knockout mice in LPS/D-GalN-induced ALI. Survival analysis showed that 85.7% of *Rnf115*^+/+^ mice were dead 6 h after intraperitoneal administration of LPS/D-GalN, whereas only 28.6% of *Rnf115*^−/−^ mice were dead at 8 h post-treatment. Approximately 71.4% of *Rnf115*^−/−^ mice were alive at 12 h post-treatment (Fig. 1a). Examination of the gross morphology of the liver showed that *Rnf115*^*+/+*^ livers displayed hemorrhaging and congestion, whereas *Rnf115*^−/−^ livers exhibited partial hemorrhaging at 5 h (Fig. 1b). The levels of serum aminotransaminase (ALT) and aspartate transaminase (AST) in *Rnf115*^−/−^ mice at 5 h post-treatment were significantly lower than those in *Rnf115*^+/+^ mice (Fig. 1c). Data from Hematoxylin and eosin (H&E) staining suggested that compared with *Rnf115*^+/+^ mice, *Rnf115*^−/−^ liver displayed less hemorrhaging and structural disorders, which were consistent with the decreased levels of ALT and AST in LPS/D-Gal-treated *Rnf115*^−/−^ mice (Fig. 1d). These results suggest that the deletion of *Rnf115* alleviated the severity of ALI in mice.

Next, we performed reciprocal *Rnf115* gain-of-function experiments using an AAV8-Rnf115 construct or AAV8-Null control. At 4 weeks following the AAV8-Rnf115 injection, the LPS/D-GalN-induced ALI mouse model was generated. As shown in Fig. 1e, compared with AAV8-Null-injected *Rnf115*^−/−^ mice, *Rnf115*^−/−^ mice in the AAV8-Rnf115 group showed more severe hemorrhaging and congestion in the liver and disrupted liver architecture (Fig. S4). Simultaneously, the recovery of Rnf115 expression in *Rnf115*^−/−^ mice increased the levels of serum ALT and AST (Fig. 1f), indicating that RNF115 promotes LPS/D-GaIN-induced ALI in mice.

### *Rnf115* knockout attenuates inflammatory response in ALI mice

**Fig. 2:**
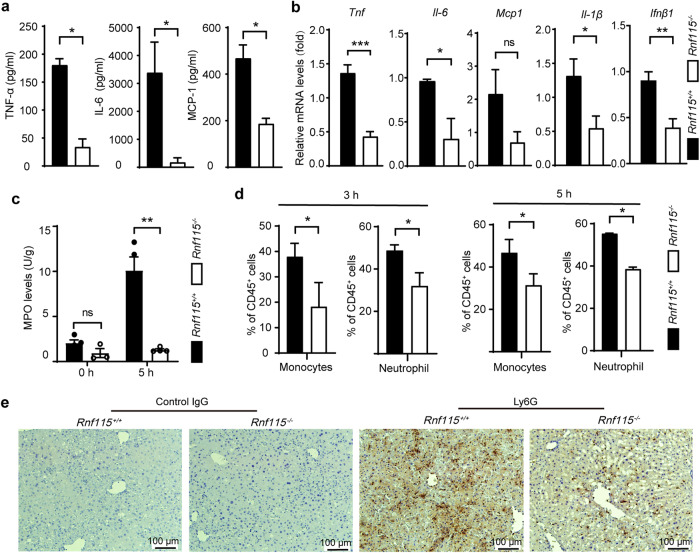
*Rnf115* knockout decreases hepatic inflammation in ALI mice induced by LPS/D-GaIN. **a** The levels of TNF, IL-6, and MCP1/CCL2 in serum with different mice were detected by LEGENDplex™ mouse proinflammatory chemokine panel. **b** The levels of *Tnf*, *Il-6*, *Mcp1/Ccl2, Il-1β and Ifnβ1* mRNA in the livers of different mice were measured by qRT–PCR. **c** The levels of MPO in the livers of different mice were detected. **d** The percentage of CD11b^+^CD14^+^ monocytes and CD11b^+^Ly6G^+^ neutrophils in livers were analyzed by Flow cytometry. **e** Ly6G^+^ cells were detected by immunohistochemical analysis. Isotype IgG staining was used as a negative control. *p < 0.05, **p < 0.01, ns: no significance.

LPS is a common endotoxin that binds and activates TLR4 and the downstream cascade signaling pathways in macrophages, leading to the release of pro-inflammatory cytokines such as TNF-α and IL-6, which promote inflammatory impairment [26]. To determine the effect of *Rnf115* deficiency on the induction of the inflammatory response, serum samples and liver tissues were harvested at 5 h following LPS/D-GaIN administration. Our findings showed that the levels of inflammatory cytokines (TNF-α and IL-6) and chemokine MCP1/CCL2 was significantly downregulated in the serum of *Rnf115*^−/−^ mice compared with those in *Rnf115*^+/+^ mice (Fig. 2a). Consistent with these findings, the levels of these cytokines (including Il-1β and Ifnβ1) in the liver tissue were lower in *Rnf115*^−/−^ mice than in *Rnf115*^+/+^ mice (Fig. 2b). Moreover, the deletion of *Rnf115* significantly decreased the activity of hepatic myeloperoxidase (MPO; Fig. 2c). Furthermore, we analyzed the degree of inflammatory cell infiltration using flow cytometry and immunohistochemistry, which indicated that the proportion of CD11b^+^CD14^+^ monocytes and CD11b^+^Ly6G^+^ neutrophils was significantly lower in *Rnf115*^−/−^ mice than in *Rnf115*^+/+^ mice, both at 3 h and 5 h after LPS/D-GalN treatment (Fig. 2d and Fig. S5). Immunohistochemical staining further confirmed the lower neutrophil infiltration in the livers of *Rnf115*^−/−^ mice treated with LPS/D-GalN (Fig. 2e). Collectively, these findings demonstrate that *Rnf115* knockout attenuated LPS/D-GaIN-induced ALI, which was associated with a decrease in the hepatic inflammation response.

### *Rnf115* knockout upregulates hepatocyte autophagy and attenuates mitochondrial damage and apoptosis

**Fig. 3:**
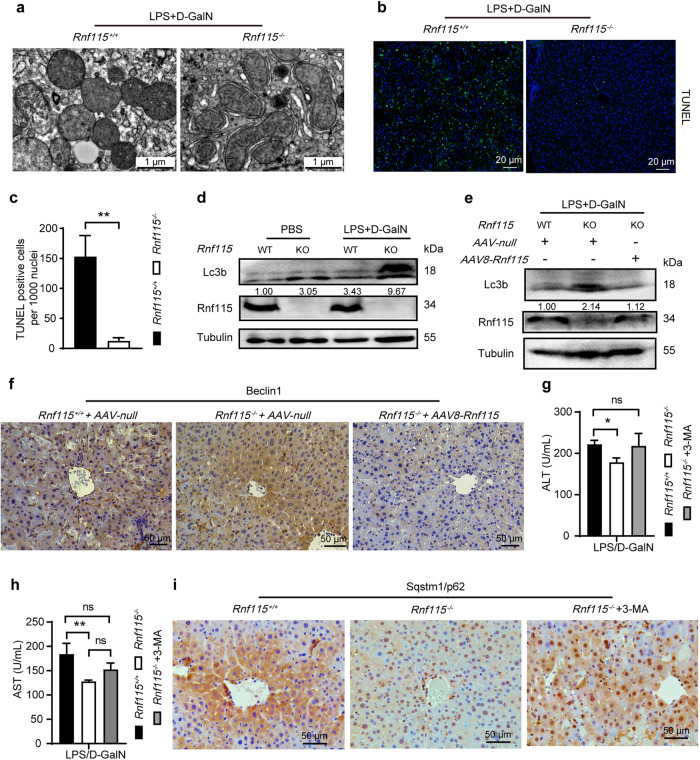
*Rnf115* knockout upregulates hepatocyte autophagy, decreases mitochondria damage and apoptosis in ALI mice. **a** Representative ultrastructural images of the liver in different mice with LPS/D-GalN treatment for 5 h by TEM. **b** Representative images of TUNEL (green) and Hoechst staining (blue) of the nuclei in the livers of different mice. **c** Quantification of TUNEL-positive cells per 1000 nuclei. **d** and **e** Representative Western blot of Lc3b and Rnf115 in the liver tissue extracts obtained from different ALI mice. **f** The expression of Beclin1 protein in liver tissues was detected by immunohistochemistry in different ALI mice. **g** Serum ALT and (**h**) AST levels from *Rnf115*^*+/+*^ and *Rnf115*^*-/-*^ ALI mice with or without 3-MA (30 mg/kg) pre-treatment for 2 h. **i** The expression of Sqstm1/p62 in different mouse livers detected by immunohistochemistry. ns: no significance, *p < 0.05, **p < 0.01. Data are representative of three independent experiments.

Using transmission electron microscopy, we analyzed the liver mitochondrial morphology in mice with ALI. As seen in Fig. 3a, *Rnf115*^+/+^ mice showed several swollen mitochondria in the liver tissue, whereas *Rnf115*^−/−^ mice showed fewer damaged mitochondria. Next, we performed a terminal deoxynucleotidyl transferase-mediated dUDP nick-end labeling (TUNEL) assay to investigate hepatocyte apoptosis in LPS/D-GaIN-induced ALI. The results showed a significantly lower proportion of apoptotic hepatocytes in *Rnf115*^−/−^ mice than in *Rnf115*^+/+^ mice (Figs. 3b and c). These data suggest that *Rnf115* deficiency decreases mitochondrial damage and hepatocyte apoptosis in mice treated with LPS/D-GaIN.

Accumulating evidence suggests that enhanced autophagy protects against liver injury [11–14]. Next, we assessed cell autophagy in *Rnf115*^+/+^ and *Rnf115*^−/−^ mice with ALI. Western blotting analysis showed that the accumulation of Lc3b in the liver was higher in *Rnf115*^−/−^ mice with ALI than in *Rnf115*^+/+^ mice (Fig. 3d**)**. However, restoration of Rnf115 expression in mouse liver achieved through AAV8-*Rnf115* injection decreased the elevated Lc3b levels in *Rnf115*^−/−^ mice (Fig. 3e). The expression pattern of BECN1/Beclin-1 protein detected by immunohistochemistry was similar to that of Lc3b (Fig. 3f). Furthermore, we injected 3-methyladenine (3-MA, an inhibitor of PIK3C3 complex and autophagy) into *Rnf115*^−/−^ mice to inhibit autophagy. The serum AST and ALT levels in these mice were comparable with those in *Rnf115*^+/+^ mice (Fig. 3g and h). Moreover, the levels of P62/SQSTM1 in *Rnf115*^−/−^ mice were lower than those in *Rnf115*^*+/+*^ mice, and the 3-MA treatment increased the expression of P62/SQSTM1 in *Rnf115*^−/−^ mice (Fig. 3i), suggesting that 3-MA-inhibited autophagy promotes liver injury induced by LPS/D-GaIN. These findings indicate that *Rnf115* deficiency-mediated autophagy contributes to the suppression of liver inflammation and maintains mitochondrial homeostasis in the context of ALI.

### RNF115 interacts with LC3B and negatively regulates its expression

**Fig. 4:**
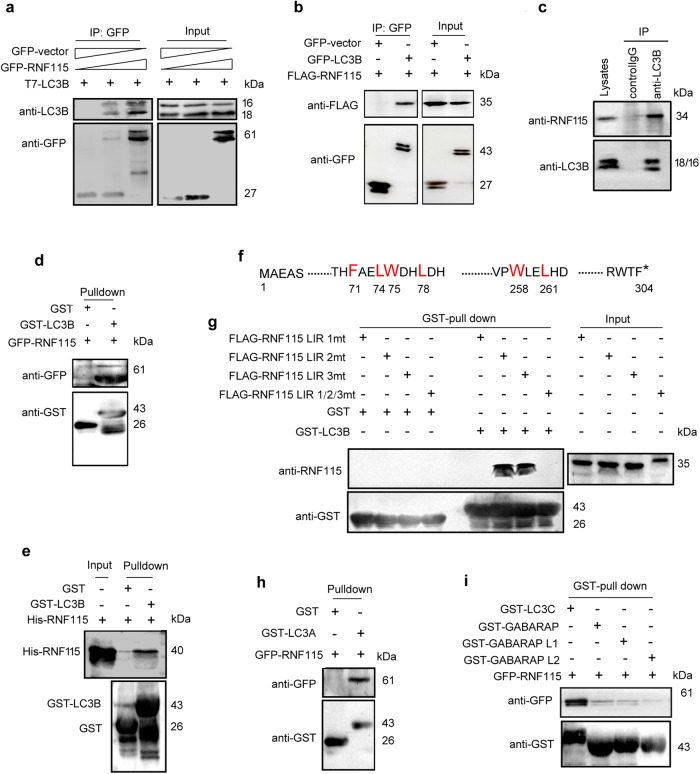
RNF115 interacts with LC3B via FAEL_71-74_ LIR domain. **a**, **b** HEK293T cells were co-transfected with indicated plasmids for 24 h. Cell lysates were subjected to IP using an anti-GFP, and indicated proteins were detected in the immunoprecipitates by western blotting. Simultaneously, 10% cell lysates were used for immunoblotting. **c** HEK293T cells were incubated with MG132 for 4 h and EBSS for 30 min, then cell lysates were subjected to IP using an anti-LC3B or a control IgG. The endogenous RNF115 and LC3B proteins were detected in the immunoprecipitates by western blotting. **d** Recombinant GST-LC3B fusion protein and the GST protein were purified and immobilized on Glutathione-Sepharose beads, then incubated with HEK293T cell lysates containing GFP-RNF115. Proteins retained on Glutathione-Sepharose were then blotted using the indicated antibodies. **e** Recombinant GST-LC3B fusion protein and the GST protein were purified and immobilized on Glutathione-Sepharose beads, then mixed with His-RNF115. Proteins retained on Glutathione-Sepharose were then blotted using the indicated antibodies. **f** Schematic diagram of the LIR domain of RNF115 protein. **g** Recombinant GST-LC3B fusion protein and the GST protein were purified and immobilized on Glutathione-Sepharose beads, then incubated with HEK293T cell lysates containing different FLAG-RNF115mutants. Proteins retained on Glutathione-Sepharose were then blotted using the indicated antibodies. **h**, **i** Recombinant GST-tagged different proteins and the GST protein were purified and immobilized on Glutathione-Sepharose beads, then incubated with HEK293T cell lysates containing GFP-RNF115. Proteins retained on Glutathione-Sepharose were then blotted using the indicated antibodies.

RNF115 acts as an E3 ubiquitin ligase with a wide range of substrates [18]; furthermore, in the present study, *Rnf115* knockout increased the levels of Lc3b. Therefore, we investigated whether LC3B is a substrate of RNF115. Co-immunoprecipitation (CO-IP) experiments showed that the T7-LC3B protein was present in the GFP-RNF115 immunoprecipitates and in a dose-dependent manner (Fig. 4a). In reciprocal co-immunoprecipitation assays, FLAG-RNF115 was detected in the GFP-LC3B immunoprecipitants (Fig. 4b), demonstrating that the two proteins interact in a complex in cells. The interaction of the endogenous RNF115 and LC3B was observed both in EBSS-incubated HEK293T cells (Fig. 4c) and mouse B16F10 cells (Fig. S6). Pull-down experiments showed that the GST-LC3B protein bound to both eukaryotic-expressed GFP-RNF115 (Fig. 4d). and prokaryotic-expressed His-RNF115 (Fig. 4e).

It is known that LC3-binding proteins have the LC3-interacting region (LIR) motif [27]. We found that the RNF115 protein contained three LIR motifs: FAEL_71-74_, WDHL_75-78_, and WLEL_258-261_, respectively (Fig. 4f). Therefore, we constructed three mutant LIR domains to determine which RNF115 mutant failed to bind LC3B. These RNF115 mutants were as follows: RNF115-LIR_1mt_ for F71/L74A, RNF115-LIR_2mt_ for W75/L78A, RNF115-LIR_3mt_ for W258/L261A, and RNF115-LIR_1/2/3mt_ for F71/L74/W75/L78/W258/L261A. The pull-down results showed that GST-LC3B interacted with RNF115-LIR_2mt_ and RNF115-LIR_3mt_ but failed to bind both RNF115-LIR_1mt_ and RNF115-LIR_1/2/3mt_ (Fig. 4g), indicating that RNF115-LIR1 is required to bind LC3B. We also studied other members of the LC3/GABARAP family. The results revealed that GST-LC3A and GST-LC3C also interacted with GFP-RNF115, whereas GABARAP, GABARAP-L1, and GABARAP-L2 did not bind GFP-RNF115 (Fig. 4h and i).

**Fig. 5:**
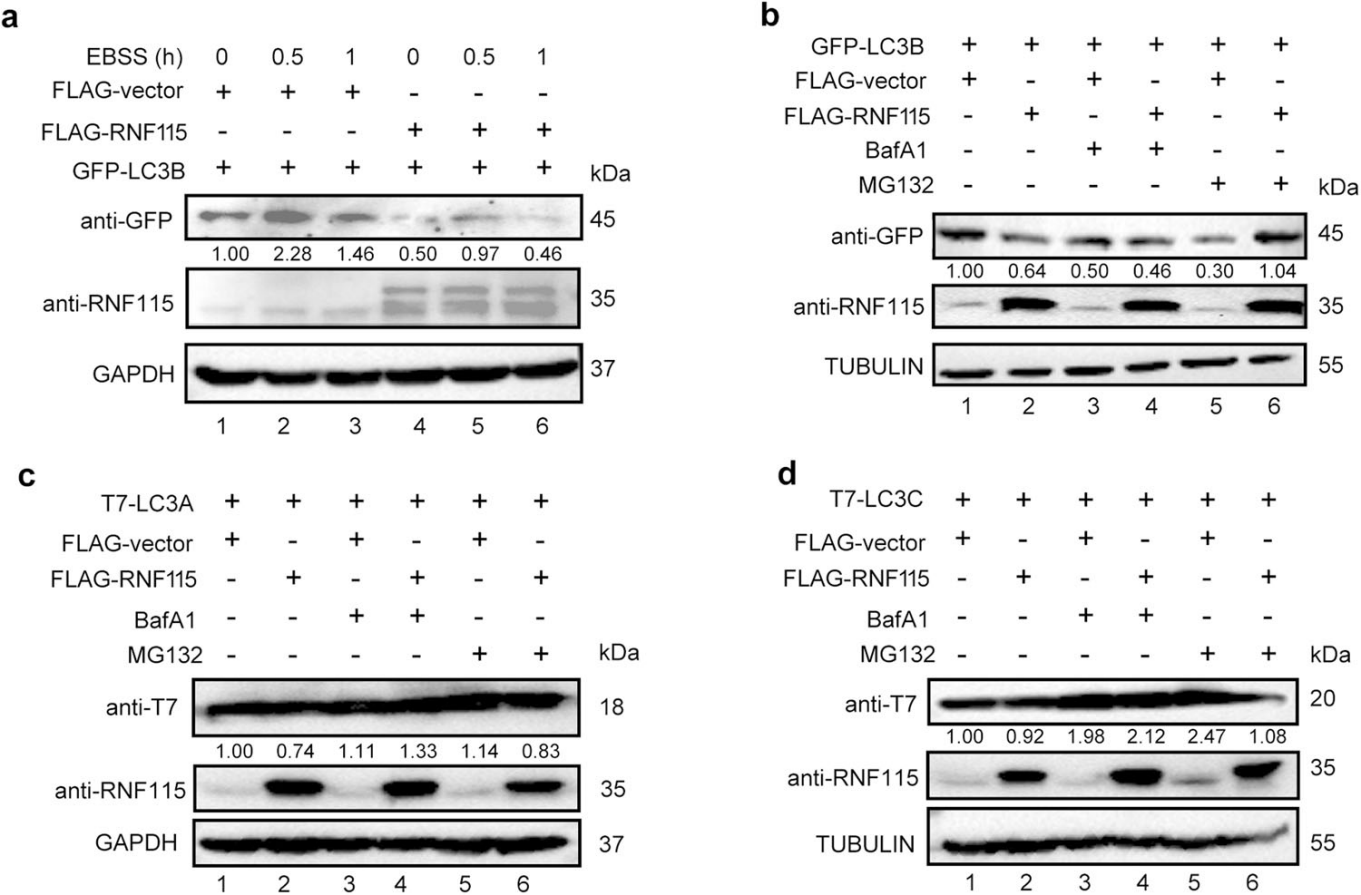
RNF115 negatively regulates LC3B protein homeostasis. **a** HEK293T cells were co-transfected with indicated plasmids for 24 h, with or without EBSS for another 0.5 h or 1 h. Western blot analysis of indicated protein in cell lysates. **b**–**d** HEK293T cells were co-transfected with indicated plasmids for 24 h, with or without BafA1 (10 μM) or MG132 (20 μM) for another 4 h. Western blot analysis of indicated protein in cell lysates.

Next, we investigated the biological significance of RNF115-LC3 interaction. Our results showed that RNF115 overexpression significantly decreased the LC3B protein levels with or without autophagic state (Fig. 5a). We then tested whether LC3B reduction in RNF115-overexpressing cells may be attributed to increased proteasomal or lysosomal degradation. As shown in Fig. 5b, the decreased LC3B was not restored in cells treated with BafA1 (lysosomal inhibitor; Fig. 5b, Lane 4 vs. Lane 3). In contrast, treatment with MG132 (proteasomal inhibitor) largely restored the LC3B expression in RNF115-overexpressing cells (Fig. 5b, Lane 6 vs. Lane 5), indicating that RNF115 might affect proteasomal degradation of LC3B. The same experiment was performed to assess LC3A and LC3C levels, and the results showed that RNF115 overexpression did not affect their levels with or without BafA1 and MG132 treatment (Fig. 5c and d).

Next, we analyzed the half-life of the LC3 protein using the protein translation inhibitor cycloheximide (CHX, 100 µg/mL). As shown in Fig. S7a and S7b, there was no net difference in LC3B decay speed in RNF115 overexpressed cells. We then tested the levels of *LC3B* transcripts. As shown in Fig. S7c and S7d, the levels of *LC3B* mRNA from the real-time quantitative PCR results were not significantly changed either in cells with RNF115 overexpression or RNF115 knockdown, indicating that RNF115 may negatively regulate LC3B expression in post-translational modification.

### *Rnf115* knockout inhibits LPS-stimulated macrophage activation

Liver macrophages play an important role in inflammatory damage in ALI. Because *Rnf115* expression is silenced in all cells of *Rnf115*^*−/−*^ mice, we wanted to investigate whether macrophages are involved in *Rnf115*-mediated effects. *Rnf115*^+/+^or *Rnf115*^−/−^ mice were intravenously injected with clodronate liposomes, and hepatic F4/80^+^ macrophages were analyzed by immunohistochemistry at 48 h post-treatment. As shown in Fig. S8a, liposome administration significantly decreased the number of F4/80^+^ macrophages in mouse liver, indicating effective clearance of mouse macrophages. Subsequently, mice were subjected to LPS/D-GalN stimulation for 5 h. As illustrated in Fig. S8b, depletion of macrophages reduced the difference in the extent of liver injury between *Rnf115*^*+/+*^ and *Rnf115*^*−/−*^ mice, demonstrating that macrophage elimination increased LPS-stimulated inflammatory damage in *Rnf115*^*–/–*^ mice. These results suggest that macrophages were required for the decrease in liver injury in LPS/D-GalN-treated *Rnf115* KO mice. Therefore, we further analyzed the biological activities of *Rnf115*^−/−^ macrophages.

**Fig. 6:**
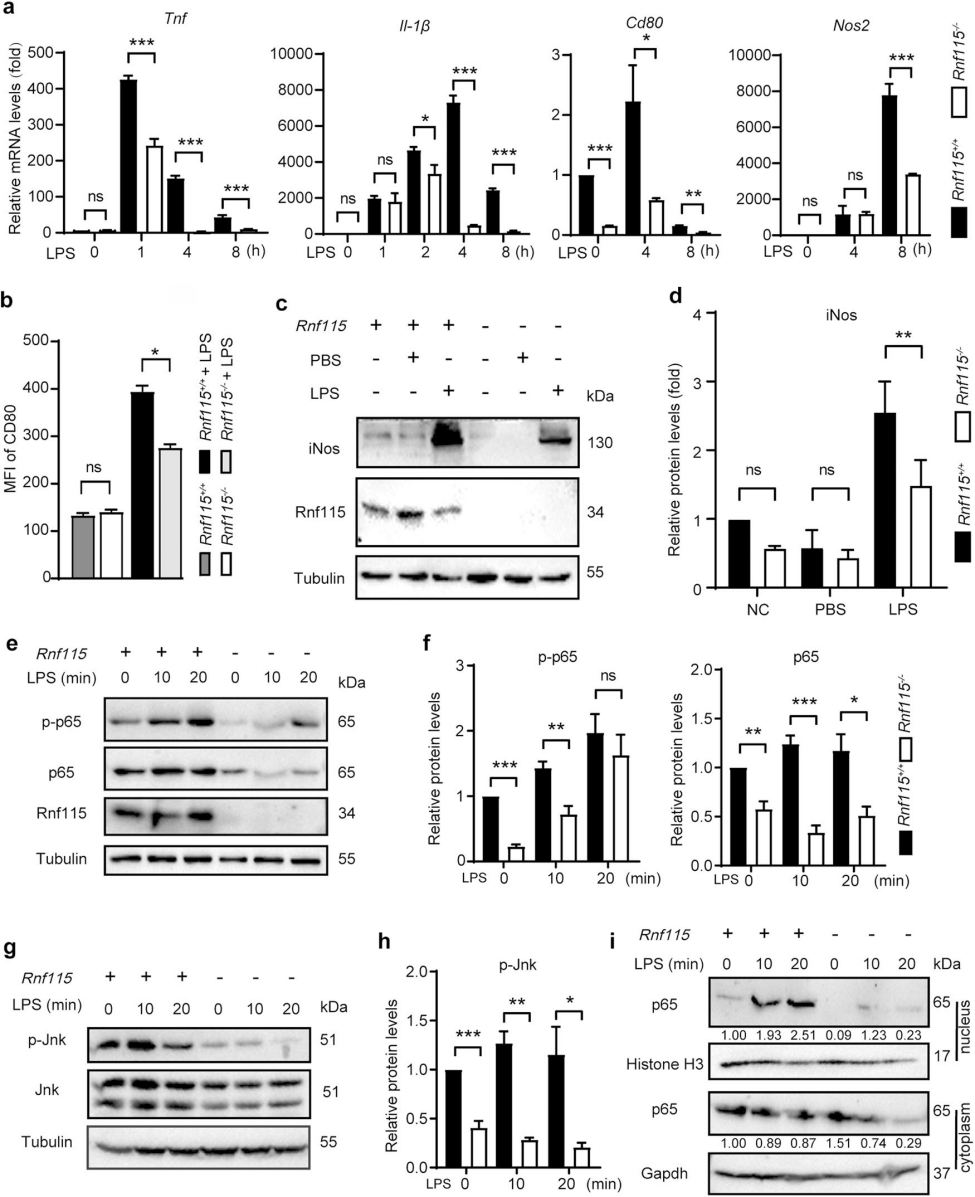
*Rnf115* knockout inhibits LPS-stimulated macrophage activation. **a** *Rnf115*^*+/+*^and *Rnf115*^*-/-*^ BMDMs were treated with or without 100 ng/ml of LPS for the indicated time, and the levels of *Tnf, Il-1β, Cd80*, and *Nos2* mRNA were measured by qRT–PCR. **b** *Rnf115*^*+/+*^and *Rnf115*^*-/-*^BMDMs were treated with or without 100 ng/ml of LPS for 24 h. The levels of CD80 were detected by flow cytometry, and the mean fluorescence intensity (MFI) was statistically analyzed. **c** *Rnf115*^*+/+*^and *Rnf115*^*-/-*^BMDMs were treated as same as **b**, then western blot analysis of indicated proteins in cell lysates. **d** Quantification of indicated proteins (ratio to TUBULIN) in cells treated as in (**c**). The average value of *Rnf115*^*+/+*^mice without LPS was normalized to 1. **e** and **g** *Rnf115*^*+/+*^and *Rnf115*^*-/-*^BMDMs were treated with or without 100 ng/ml of LPS for the indicated time. Immunoblot analysis of indicated proteins in cell lysates. **f** and **h** Quantification of indicated proteins (ratio to TUBULIN) in cells treated as in (**e** and **g**). The average value of *Rnf115*^*+/+*^mice without LPS was normalized to 1. **i** *Rnf115*^*+/+*^and *Rnf115*^*-/-*^BMDMs were treated with or without 100 ng/ml of LPS for the indicated time. Immunoblot analysis of indicated proteins in the nucleus and cytoplasm. *p < 0.05, **p < 0.01, ***p < 0.001, ns: no significance.

BMDMs isolated from the bone marrow of *Rnf115*^+/+^ and *Rnf115*^−/−^ mice were used as experimental cells. qRT-PCR results showed that the mRNA levels of *Tnf-α*, *Il-1β*, *Cd80*, and *Nos2* were significantly lower in *Rnf115*^−/−^ BMDMs than that in *Rnf115*^+/+^ BMDMs after stimulation with LPS (Fig. 6a). Moreover, flow cytometry data (Figs. S8c and 6b) and western blotting (Figs. 6c and d) also showed that the protein levels of Cd80 and iNOS were lower in *Rnf115*^−/−^ BMDMs. These findings suggest that *Rnf115* KO reduced the activation of M1 macrophages and the inflammatory response to LPS.

LPS-stimulated macrophages activate the NF-κB and MAPK pro-inflammatory signaling pathways to induce the release of inflammatory cytokines. To investigate the potential mechanism underlying RNF115-regulated macrophage inactivation, *Rnf115*^+/+^ and *Rnf115*^–/–^ BMDMs were stimulated with 100 ng/mL LPS for the indicated times. The levels of total and phosphorylated NF-κB p65 and MAPKs were then assessed by immunoblotting. Compared with LPS-treated *Rnf115*^+/+^, LPS-treated *Rnf115*^–/–^ BMDMs showed a significant decrease in the levels of total and phosphorylated NF-κB p65 (Figs. 6e and f) and phosphorylated Jnk (Figs. 6g and h). However, *Rnf115* KO did not affect the activity of ERK1/2 and p38 MAPK (Fig. S9).

Because the nuclear translocation of NF-κB p65 is a key indicator of the activation of NF-κB signaling pathway, we next examined the distribution of the NF-κB p65 subunit using confocal microscopy. The fluorescence intensity and nuclear distribution of NF-κB p65 subunit were significantly lower in *Rnf115*^–/–^ macrophages than that in *Rnf115*^*+/+*^ macrophages (Fig. S10). Using the nucleoplasm separation kit, we further demonstrated that the nuclear accumulation of p65 protein in *Rnf115*^−/−^ BMDMs was significantly lower than that in *Rnf115*^+/+^ BMDMs after LPS stimulation (Fig. 6i). Collectively, the above data indicate that for LPS stimulation, *Rnf115* KO limits the NF-κB and Jnk signaling pathways, consequently inhibiting macrophage activation and the release of inflammatory cytokines. These results indicate that *Rnf115* deficiency-mediated inactivation of macrophages contributes to the suppression of liver inflammatory injury in the context of ALI.

## Discussion

In the present study, we investigated the regulatory role of RNF115/BCA2 in *Rnf115* knockout mice with LPS/D-GalN-induced ALI. The results demonstrated that *Rnf115* KO mice with ALI displayed prolonged survival time and reduced liver damage, accompanied by increased autophagy in the liver and the inhibition of inflammatory response. Further experiments indicated that RNF115 interacts with LC3B, negatively regulates LC3B protein levels and cell autophagy. Additionally, the protective effect of *Rnf115* KO may have contributed to the inactivation of macrophages via the downregulation of NF-κB and Jnk signaling pathways, leading to decreased production of pro-inflammatory cytokines (TNF-α and IL-6) and chemokines CCL2 and iNOS, consequently ameliorating LPS/D-GalN-induced inflammatory damage.

Accumulating evidence has indicated that autophagy, a lysosome-mediated cellular catabolic process, plays an important role in maintaining the dynamic balance of hepatic cells and metabolism. Dysfunction of autophagy is associated with various liver diseases, including liver injury, hepatitis, and hepatocellular carcinoma [28, 29]. Administration of LPS/D-GalN is a well-established and widely used approach to induce ALI in mice. The mechanism of ALI induced by LPS/D-GalN is as follows: (1) D-GalN mediates the sensitivity of hepatocytes to LPS-induced cytotoxicity and (2) LPS activates macrophages to produce pro-inflammatory cytokines such as TNF-α, which causes hepatocyte apoptosis or death. In combination, these effects trigger ALI in mice. Earlier studies have shown that autophagy protects hepatocytes from TNF-α-induced liver injury. In hepatocyte-specific *Atg7* KO mice, serum alanine aminotransferase levels were elevated in LPS/D-GalN-treated mice. Defective autophagy increased the sensitivity of hepatocytes to LPS/D-GalN, leading to increased tissue damage, apoptosis, and cell death. Increased autophagy owing to Beclin1 overexpression prevents LPS/D-GalN-induced liver injury. Autophagy inhibits TNF toxicity by blocking caspase 8 activation and mitochondrial death pathways in vivo, suggesting that autophagy is a potential therapeutic target in the treatment of TNF-α-induced liver injury [30]. Fibroblast growth factor 21 was recently found to attenuate CCl4-induced ALI through SIRT1-specific mediated autophagy-induced expression [31]. A previous study in our laboratory showed that enhanced autophagy accelerated the clearance of damaged mitochondria in drug-induced ALI in mice, thus providing a protective effect [14]. In contrast, some studies have shown that the autophagy inhibitor wortmannin attenuates lipopolysaccharide-induced acute hepatitis [32]; thus, the role of autophagy in liver injury remains somewhat controversial. In the present study, we found that *Rnf115* knockout significantly attenuated liver injury and significantly prolonged the survival of mice through autophagy in mice with ALI. However, the autophagy inhibitor 3-MA exacerbated liver damage in *Rnf115*^*-/-*^ mice with ALI, further supporting that autophagy is a protective mechanism in ALI. Therefore, promoting autophagy is a potential therapeutic strategy to alleviate ALI.

LC3 is closely associated with and essential for autophagosome formation and maturation. In mammals, there are at least seven LC3/GABARAP family members: LC3A-a and LC3A-b, LC3B, LC3C, GABARAP, GABARAPL1, and GABARAPL2. The most studied of these proteins is LC3B, which is used to monitor autophagic activity. Given the high homology, the other LC3/GABARAP members are often presumed to have a similar function. The molecules bound to LC3 contain one or several LIR motifs [27]. The sequencing analysis of the RNF115 protein revealed that it contains three LIR motifs (Fig. 4e). It was found that the interaction of RNF115 with LC3B is dependent on the FAEL_71-74_ LIR domain. RNF115 also binds to LC3A and LC3C proteins but not to GABARAP family proteins. Further experiments revealed that RNF115 downregulated LC3B protein levels but had no significant effect on LC3A and LC3C. Since RNF115 failed to affect the transcript level of LC3B (Fig. S7c and S7d), we propose that RNF115 may function on the post-translational modification of LC3B. However, we cannot exclude other mechanisms for regulating LC3B, which needs to be further explored. Thus, RNF115 negatively regulates cell autophagy mainly through LC3B but not through other family members. Our study adds a new dimension to the fine regulatory network of LC3B.

LPS is the main ligand for TLR4 on the surface of macrophages. Activated LPS–TLR4 complex induces the activation of the MyD88-dependent and MyD88-independent pathways [33, 34]. Subsequently, MyD88 binds to the IRAKs/TRAF6 and TAK1/TABs complex, leading to the activation of IKK. Then, IKK phosphorylates IκBα, which stimulates the nuclear translocation of NF-κB, which in turn induces macrophages to produce pro-inflammatory factors such as TNF-α, IL-6, IL-1, and MCP1/CCL2. Such excessive or unresolved M1 macrophage activation leads to acute/chronic inflammation and tissue damage. Recent studies suggest that *Rnf115* knockout promotes the translocation of TLRs from the endoplasmic reticulum to the Golgi apparatus and then to the lysosomes and cell membrane, leading to greater resistance to bacterial infection in *Rnf115* knockout mice [25]. The present study indicates that *Rnf115* knockout downregulated the levels of Tnf-α, Il-6, Il-1β and Ifnβ1 in liver tissues induced by LPS/D-GalN. Furthermore, *Rnf115*^−/−^ macrophages showed lower sensitivity to LPS stimulation, as evidenced by the decrease in NF-κB and JNK signaling and the release of TNF-α, IL-6, and CCL2, accompanied by the downregulation of the levels of Cd80 and iNOS in *Rnf115*^−/−^ macrophages, which suggests the inactivation of M1 macrophages. Depletion of macrophages exacerbated the liver damage, indicating that macrophages were required for the reduction in inflammatory damage in LPS/D-GalN-treated *Rnf115*^*-/-*^ mice. Therefore, we deduced that in the liver of LPS/D-GalN-treated *Rnf115*^−/−^ mice, such effects may protect against acute liver damage by attenuating the infiltration of monocytes and neutrophils and TNF-α-induced liver cell apoptosis and death. However, further research is needed to elucidate the precise molecular mechanism by which RNF115 regulates macrophage activation.

We note that the modulatory effect of RNF115 on the NF-κB signaling pathway is controversial, stating both pro- and anti- NF-κB activity. A previous report suggests that RNF115/BCA2 negatively regulates the NF-κB pathway in 293 T cells treated by viral infection [35]. Shi et al. [36] reveals that overexpression of RNF115 decreased NF-κB activation in HEK293T cells and breast cancer cells (MCF-7, MDA-MB-231) triggered by TNF-α stimulation. However, the depletion of RNF115 reduced the basal activation levels of NF-κB in non-tumor MCF-12F cells [36]. Consistently, our data indicate that *Rnf115* KO in BMDM can limit the NF-κB signaling pathway after LPS stimulation (Figs. 2b and 6a-d). We speculate that RNF115 may exert a cell-type-specific effect on the regulation of NF-κB pathway, depending on different cell types as well as different conditions. The regulation of RNF115 on the NF-κB and the relationship between RNF115-regulated autophagy and macrophage activation need further investigation.

## Material and methods

### Antibodies and reagents

The antibodies and major reagents used in this study are listed in Supplementary Table 1.

### Plasmid construction

The following plasmids were successfully constructed in our Lab: GFP-RNF115, FLAG-RNF115, His-RNF115, GFP-LC3B, and FLAG-RNF115 LIR mutants. T7-LC3A, T7-LC3B, and T7-LC3C were kindly provided by Liang Ge (Tsinghua University). GST-LC3A, GST-LC3B, GST-LC3C, GST-GABARAP, GST-GABARAPL1, and GST-GABARAPL2 were kindly provided by Wei Liu (Zhejiang University). All plasmids were confirmed by DNA sequencing.

### *Rnf115* gene KO mice

*Rnf115* KO mice of C57BL/6 background were produced using CRISPR/Cas9 genome editing with guide RNA (*sgRNA1*: 5′- CCACATGCTTAGATTCCTAATGA -3′; *sgRNA2*: 5’- CCAGGTATTGTATAAGCTTATGG -3’) targeting exon 2 of mouse *Rnf115* at Shanghai BRL Medicine Inc. Offspring from the founder containing 124 base pairs (bp) deletion genotyping was performed by PCR using oligonucleotides 5′- TGTACAGTTCAAAACCAGCTT -3′(forward) and 5′- CCACTCAGTCAACACTAAGG -3′ (reverse) [wild-type (WT) allele (651 bp), mutant allele (527 bp)].

The mutant mice appeared phenotypically normal, and no obvious developmental and reproductive defects were observed. All mice were housed in a specific pathogen free (SPF) facility at a constant room temperature with free access to water and standard mouse chow. All animal experimental procedures and techniques were approved by the Animal Ethics Committee of Peking University Health Sciences Center (LA2022406).

### Animal model

Male C57BL/6 mice (8–12 weeks old) were intraperitoneally (i.p.) injected with LPS (30 μg/kg) and D-GalN (350 mg/kg) to induce ALI. Control mice received the same volume of PBS. To inhibit autophagy, 3-MA (30 mg/kg) was given i.p. to mice 2 h before the administration of LPS/D-GalN. For macrophage depletion, mice (aged 8 weeks) were intravenously injected with clodronate liposomes (200 μl/mouse) 48 h before LPS/D-GalN administration to clear macrophages.

For the rescue assay, *AAV8-Rnf115* (Shanghai GenePharma Co., Ltd., Shanghai, China) was injected via the tail vein into mice four weeks before the injection of LPS/D-GalN, and *AAV8*-null was used as a control. The mice were killed at various time points following LPS/D-GalN treatment, and the liver tissue and serum samples were collected for future analysis.

### Culture of mouse bone marrow-derived macrophages (BMDMs)

Mouse bone marrow cells were separated and cultured in Dulbecco’s modified Eagle’s medium (DMEM), supplemented with macrophage colony-stimulating factor (M-CSF) and 10% Ausbian FCS for 7 days. BMDMs were harvested with ice-cold TEN buffer [40 mM Tris, 4 mM ethylenediamine tetraacetic acid (EDTA), 0.15 M NaCl, pH 8.0] and resuspended at 5 × 10^5^ /ml in DMEM with 10% FCS and seeded for at least 6 h before stimulation.

### Serum ALT, AST, and liver MPO detection

Serum ALT and AST were measured using commercial diagnostic kits (Applygen, Beijing, China) following the manufacturer’s instructions. Liver MPO levels were detected using diagnostic kits (Nanjing Jiancheng Bioengineering Institute, Nanjing, China, A044) following the manufacturer’s instructions.

### Quantitative real-time PCR (qRT-PCR) assays

Total RNA samples were extracted from cells or tissues with the TRIzol reagent (Invitrogen, Carlsbad, CA, USA; 15596026). qRT–PCR was performed using SYBR Green Master Mix (Vazyme, Nanjing, China, Q131-02). The primers against the indicated genes used in this study are listed in the Supporting information, Table S2. All gene expressions were normalized to β-actin/ACTB. Relative gene expression in real-time PCR was calculated as follows. First, we obtained the ΔCT value by subtracting the average CT value of the reference gene from the average CT value of the target gene. Then, the average ΔCT value in the control group was calculated and the corresponding ΔΔCT values were obtained by subtracting this average from the average value of each ΔCT value in each group. Next, the 2-ΔΔCT value was calculated. All experiments were performed in triplicate, and melting curve analysis was performed to monitor the specificity.

### Co-immunoprecipitation and Western blot analysis

The total protein from mouse livers and cells was extracted using RIPA lysis buffer (50 mM Tris [pH 7.4], 150 mM NaCl, 1% NP-40, 0.5% sodium deoxycholate, 0.1% SDS, Beyotime, Shanghai, China) containing a freshly added proteinase inhibitor cocktail (Roche Diagnostics, Berlin, Germany). For Co-Immunoprecipitation, cell lysates (600 μL) were incubated with anti-GFP affinity beads 4FF (SA07005, Smart-Lifescience, Changzhou, China) for 4 h. The immunoprecipitates were washed three times by 1 mL prelysis buffer and subject to immunoblot analysis. For normal Western blot analysis, protein concentrations were determined using a BCA protein assay reagent (Beyotime, Shanghai, China; P0010). Equal amounts of proteins were separated by SDS-PAGE electrophoresis and transferred to polyvinylirdenediflouride (PVDF) membranes at 4°C (Millipore, USA). After blocking with 5% nonfat milk for 1 h, the membranes were incubated with the primary antibodies overnight at 4°C, washed, and then incubated with the goat Anti-rabbit IgG Horseradish Peroxidase Conjugate (Bioss, Beijing, China) secondary antibodies. The membranes were then washed and the protein was visualized with enhanced chemiluminescence solution and taken image using a chemiluminescent imaging system (iBright 750, Thermo Scientific, Waltham, MA, USA). The scanned bands were quantified using ImageJ software. The results were representative of at least three experiments.

### In vitro GST-pulldown assays

Soluble recombinant GST, GST-LC3A, GST-LC3B, GST-LC3C, GST-GABARAP, GST-GABARAPL1, GST-GABARAPL2 fusion proteins were expressed in *Escherichia coli* BL21- -CodenPlus (DE3) and purified on glutathione Sepharose 4B beads (GE Healthcare, 17-0851-01). Recombinant His-RNF115 was purified on Ni-NTA agarose beads. Equal amounts of GST or GST-tagged protein were incubated with GST Beads, then mixed with the whole cell lysates extracted from the indicated plasmids transfected cells for 2 h at 4 °C. After five washes, the beads were resuspended in 2 × SDS loading buffer and analyzed by Western blotting. To examine the interaction of LC3B with RNF115, the GST or GST-LC3B-conjugated beads were incubated with HIS-RNF115 in pulldown buffer for 1 h at 4 °C. The beads were washed and the bound proteins were separated by SDS-PAGE and visualized by immunoblotting.

### Detection of cytokines

The levels of Il-6, Tnf-α, and Mcp1/Ccl2 in the serum were measured by LEGENDplex™ mouse proinflammatory chemokine panel (740451; BioLegend, San Diego, CA, USA), according to the manufacturer’s instructions.

### Histological and immunohistochemical analysis

The liver tissues were fixed overnight in 4% paraformaldehyde, dehydrated in a graded series of ethanol, and embedded in paraffin. In the histopathological analysis, 4 μm sections were stained with H&E using standard procedures.

For the immunohistochemical analysis, sections were deparaffinized and rehydrated. Antigen retrieval was performed in a pressure cooker at 100 °C for 2 min in 0.01 M sodium citrate (pH 6.0), and endogenous peroxidase activity was blocked with 3% hydrogen peroxide. The slides were then incubated in 5% goat serum. Following incubation with primary antibodies at 4 °C overnight and washing three times in PBS, the sections were conducted with a DAB Detection Kit (PV-9009, ZSGB-BIO, Beijing, China) according to the manufacturer’s instructions. The sections were developed with a DAB substrate and counter-stained with hematoxylin. The samples were then dehydrated and sealed with coverslips.

TUNEL assays were performed using an in situ cell death detection kit (Roche Applied Science, Indianapolis, IN, USA) according to the manufacturer’s instructions. The sections were counterstained with Hoechst 33342 (Sigma Aldrich, 14533).

### Cell isolation and flow cytometry

Mice were euthanized by CO^2^ asphyxiation. Liver monocytes and granulocytes were separated by collagenase IV, DNase I, and Percoll. Different cells were stained with fluorescein-labeled antibodies (Supporting information, Tables S1) and analyzed by flow cytometry (FACS Aria; BD Biosciences, San Jose, CA, USA).

### Transmission electron microscopy

The liver tissues were initially fixed in a mixture of paraformaldehyde (2%) and glutaraldehyde (2.5%) stored at 4°C and then washed 4 times with PB buffer (0.1 M). The tissue underwent post-fixation with Osmium tetroxide (1%) and Tetrapotassium hexacyanoferrate trihydrate (1.5%) for 1 h at 23°C, followed by ethanol dehydration in graded solutions (50%, 70%, 80%, 90%, 100%, 100%, 100%) for 10 min each. Then, 1, 2-Epoxypropane twice for 10 min each and gradient infiltration with a mixture of 1, 2-Epoxypropane and Epon 812 resin for 8 h (SPI, America). Subsequently, Pure Epon 812 was twice and polymerized in an oven (60°C). Blocks of polymerized resin were sectioned using a Leica EM UC7 ultramicrotome (Wetzlar, Germany). Ultra-thin sections (70 nm) were mounted and dried on coated copper grids. Sections were stained on-grid with 2% uranyl acetate (25 min) and lead citrate (5 min). Imaging was carried out using an H-7650B transmission electron microscope (Hitachi, Tokyo, Japan).

### Immunofluorescence analysis

BMDMs were seeded onto 12 mm micro cover glasses (Electron Microscopy Sciences). After LPS treatment, cells were fixed with 4% paraformaldehyde for 10 min at room temperature, rinsed with PBS, and permeabilized with 0.1% triton X-100 (Sigma) for 5 min. Thereafter, cells were blocked for 30 min with 5% BSA in PBS and consequently incubated with primary antibodies against p65 (1:500) overnight at 4 °C and Hoechst 33342 (1:10,000) for 5 min. Cells were observed under Laser confocal fluorescence microscope (LSM 880; Carl Zeiss AG, Oberkochen, Germany).

### Statistical analysis

A Gehan–Breslow–Wilcoxon test was used to compare the Kaplan–Meier survival curves between the different groups of mice generated in GraphPad Prism version 8. Unpaired Student’s t-tests (two-tailed) were performed using Prism software. A *p* value < 0.05 was considered significant.

### Supplementary information

Supplementary figures and figure legends

Supplementary Table 1

Supplementary Table 2

Original Data File

checklist

## Data Availability

The data can be made available upon reasonable request to the corresponding author at the following address: Yingyu Chen, Department of Immunology, Peking University School of Basic Medical Sciences, 38 Xueyuan Road, Beijing, 100191, China. Email: yingyu_chen@bjmu.edu.cn.
