# Supplementary material for: RNF115/BCA2 deficiency alleviated acute liver injury in mice by promoting autophagy and inhibiting inflammatory response
Source: Cell Death Dis. 2023 Dec 21;14(12):855. doi: 10.1038/s41419-023-06379-7 (PMC10739886; doi:10.1038/s41419-023-06379-7)
Supplement: Supplementary file 1 — Supplementary figures and figure legends [file 41419_2023_6379_MOESM1_ESM.pdf]

Supplementary data

RNF115/BCA2 deficiency alleviated acute liver injury in mice by promoting autophagy and inhibiting inflammatory response

Jinqiu Feng[1], Shufang Ye[1], Bao Hai[2], Yaxin Lou[3], Mengyuan Duan[1], Pengli Guo[1], Ping Lv[1], Wenping Lu[4, *], Yingyu Chen[1,5, *]

[1] Department of Immunology, Peking University School of Basic Medical Sciences; NHC Key Laboratory of Medical Immunology, Peking University, 38 Xueyuan Road, Beijing, 100191, China.

[2] Department of Orthopedics, Peking University Third Hospital, 49 North Garden Road, Beijing, 100191, China.

[3] Medical and Healthy Analytical Center, Peking University, 38 Xueyuan Road, Beijing, 100191, China.

[4] Department of Hepatobiliary Surgery, First Medical Center, Chinese PLA General Hospital, 28 Fuxing Road, Beijing, 100853, China. lvwenping301@126.com.

[5] Center for Human Disease Genomics, Peking University, 38 Xueyuan Road, Beijing, 100191, China.

*Corresponding authors. Email addresses: lvwenping301@126.com, yingyu_chen@bjmu.edu.cn

Supplementary Figures and Figure Legends

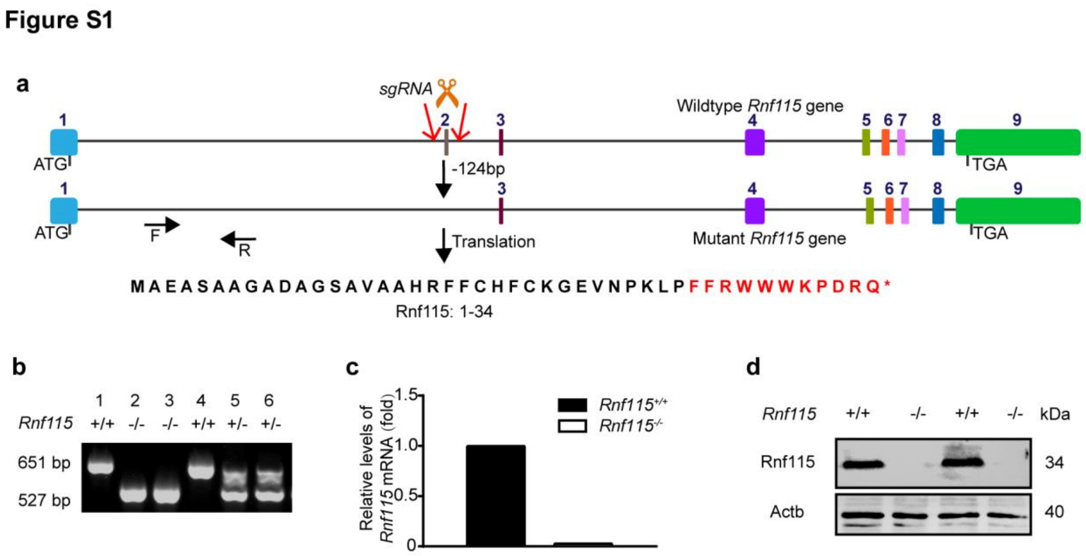

Fig. S1 CRISPR/Cas9 genome editing of mouse *Rnf115*. (a) The genomic structure of wildtype and mutant Rnf115 gene is shown. The boxes represent exons of Rnf115, and the targeting sites are shown. (b) Genomic PCR was used to identify the mutations of Rnf115gene in mice. (c) *Rnf115* mRNA levels in *Rnf115*$^{+/+}$and *Rnf115*$^{-/-}$BMDMs were measured by qRT–PCR. (d) The levels of Rnf115 protein in *Rnf115*$^{+/+}$and *Rnf115*$^{-/-}$ BMDMs were detected by Western blotting.

Figure S2

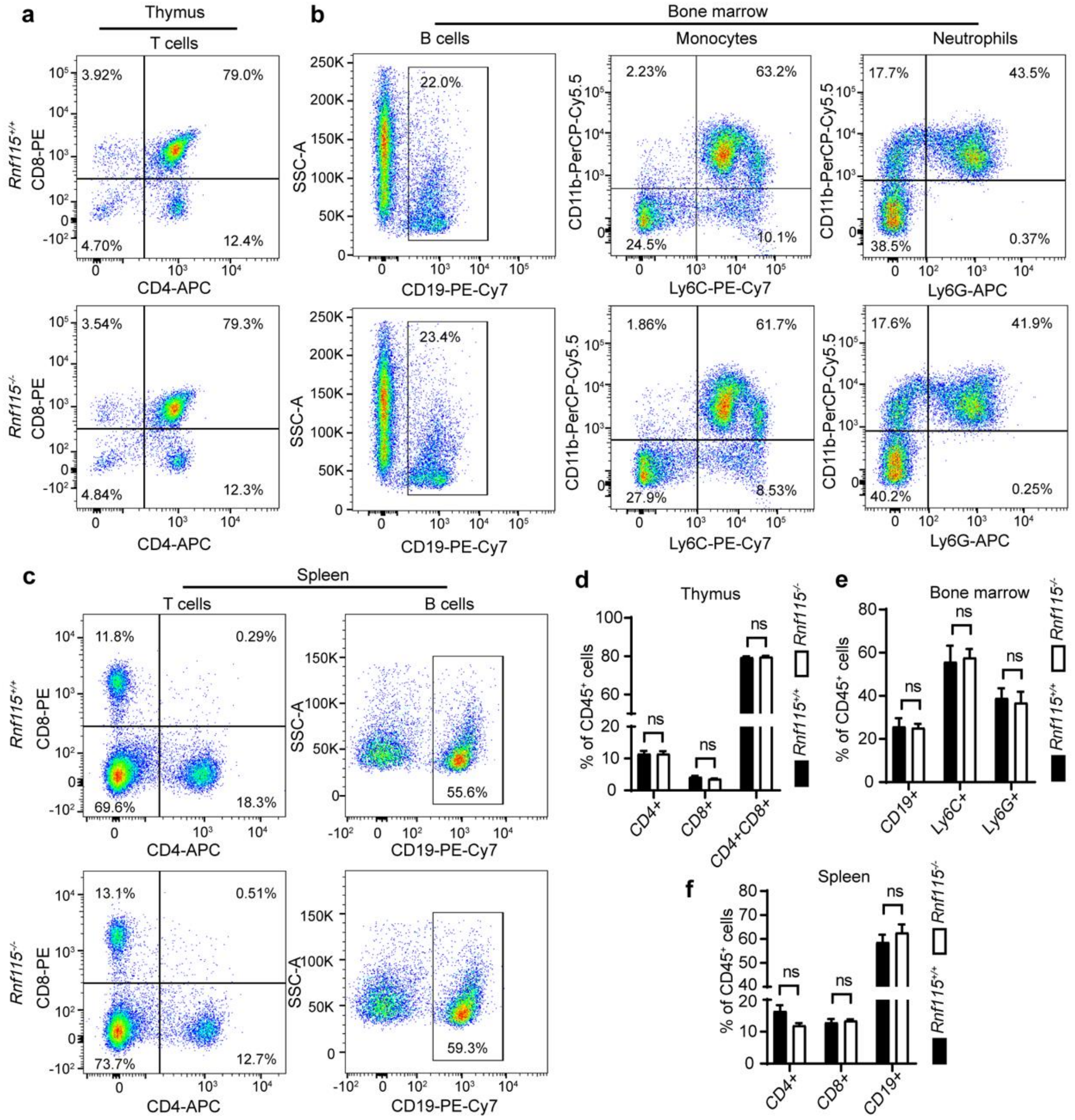

Fig. S2. ***Rnf115* knockout did not affect immunocyte development and homeostasis.**

(a-c) Flow cytometry analysis of the proportions of (a) $CD45^+CD4^+$ T cells, $CD45^+CD8^+$ T cells in thymus; (b) $CD45^+CD19^+$ B cells, $CD45^+CD11b^+Ly6C^+$ monocytes, $CD45^+CD11b^+Ly6G^+$ neutrophils in bone marrow; (c) $CD45^+CD4^+$ T cells, $CD45^+CD8^+$ T cells, $CD45^+CD19^+$ B cells in spleen between *Rnf115*$^{+/+}$ and *Rnf115*$^{–/–}$ mice. (d-f) Statistical analysis (mean ± SD, n=6).

Figure S3

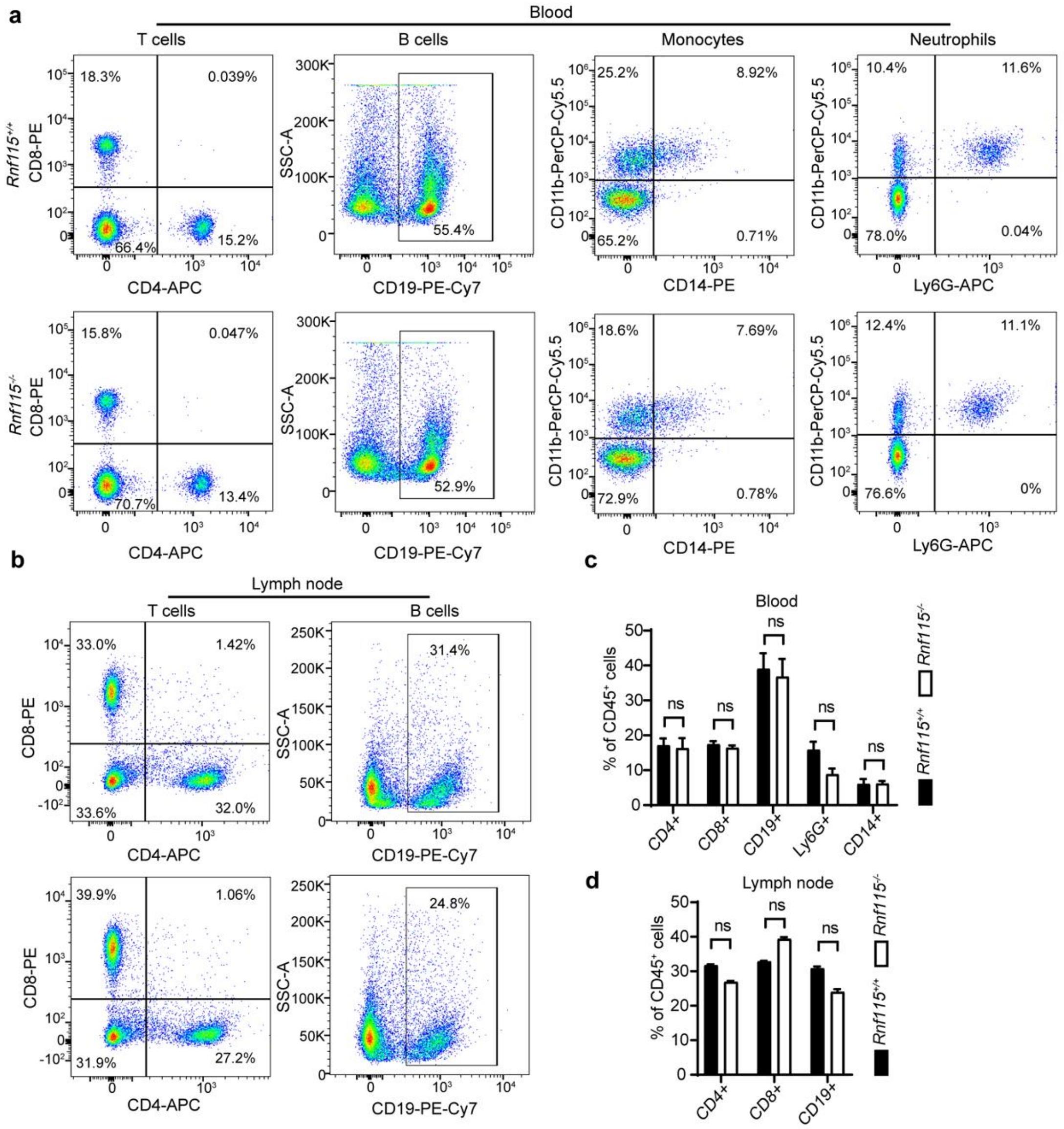

Fig. S3. ***Rnf115*** **knockout did not affect immunocyte development and homeostasis.**

(a-b) Flow cytometry analysis of the proportions of (a) $CD45^+CD4^+$ T cells, $CD45^+CD8^+$ T cells, $CD45^+CD19^+$ B cells, $CD45^+CD11b^+CD14^+$ monocytes, $CD45^+CD11b^+Ly6G^+$ neutrophils in blood; (b) $CD45^+CD4^+$ T cells, $CD45^+CD8^+$ T cells, $CD45^+CD19^+$ B cells in lymph node between *Rnf115*$^{+/+}$ and *Rnf115*$^{-/-}$ mice. (c-d) Statistical analysis (mean ± SD, n=6).

Figure S4

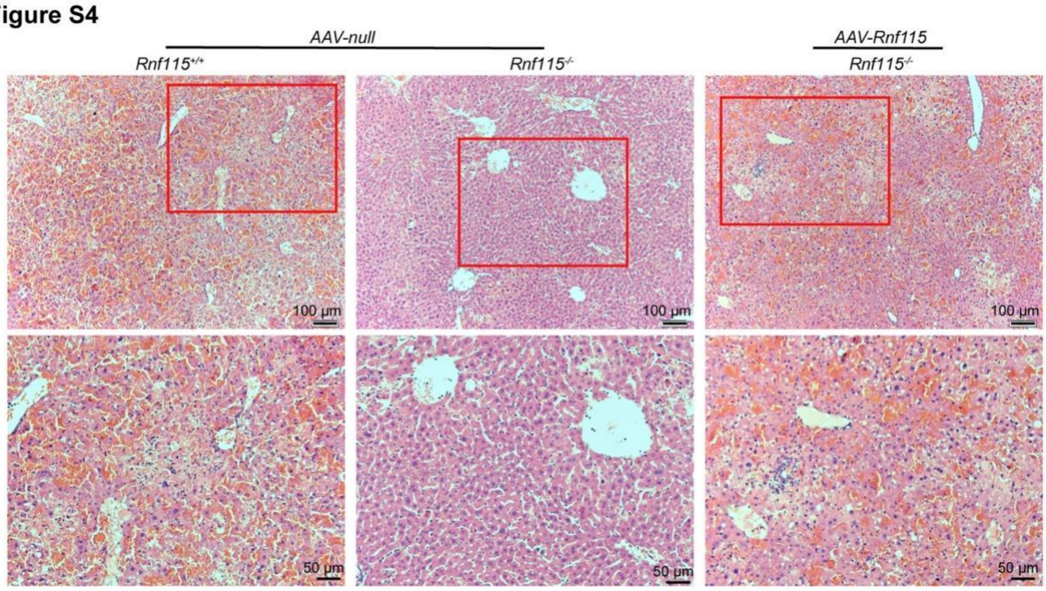

Fig. S4. RNF115 promotes LPS/D-GaIN-induced ALI in mice. Representative images of livers stained with H&E staining from different ALI mice treated by LPS/D-GalN for 5 h.

Figure S5

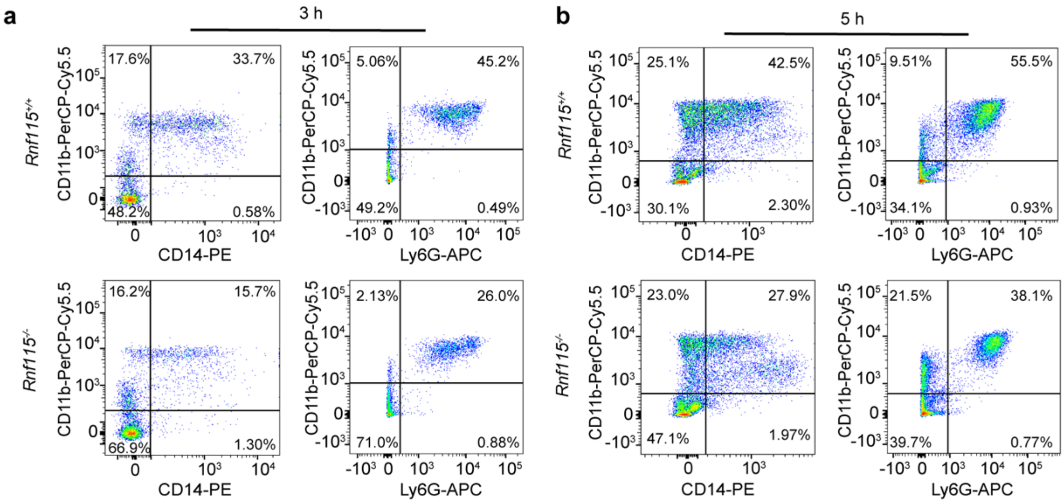

Fig. S5 Rnf115 knockout decreased the infiltration of monocytes and neutrophils in ALI liver. *Rnf115*$^{+/+}$ and *Rnf115*$^{-/-}$ mice were treated with LPS/D-GalN for (a) 3 h and (b) 5 h. The number of CD11b^{+}CD14^{+} monocytes and CD11b^{+}Ly6G^{+} neutrophils in liver CD45^{+} cells were detected by flow cytometry.

Figure S6

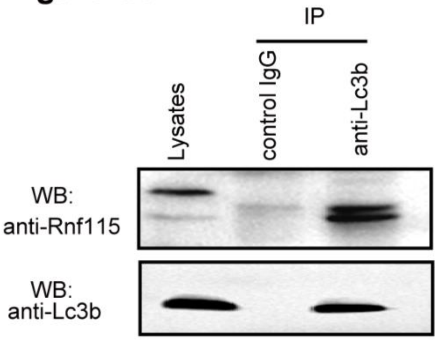

Fig. S6 Rnf115 interacts with Lc3b in mice. Mouse B16F10 cells were incubated with MG132 for 4 h, then cell lysates were subjected to IP using an anti-Lc3b or a control IgG. The endogenous Rnf115 and Lc3b proteins were detected in the immunoprecipitates by western blotting.

Figure S7

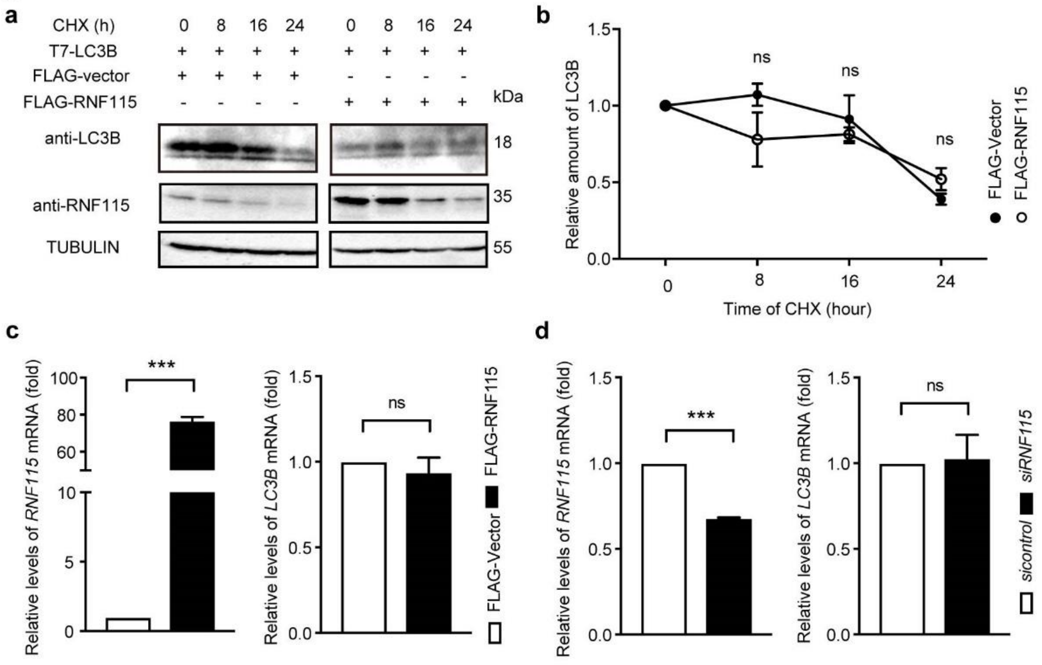

Fig. S7 RNF115 had no effect on the LC3B protein decay and *LC3B* mRNA levels. (a) HEK293T cells were cotransfected with indicated plasmids for 24 h, then incubated with cycloheximide (CHX, 50 μg/mL) for the indicated time. The levels of T7-LC3B were detected by western blotting. (b) Quantification of LC3B proteins (ratio to TUBLIN) in cells treated as in (a)**.** (c) HEK293T cells were transfected with FLAG-vectors or FLAG-RNF115 for 24 h, the levels of both *RNF115* and *LC3B* mRNA were measured by qRT–PCR. (d) HEK293T cells were transfected with non-silencing control siRNA (*sicontrol*) or siRNA against RNF115 (*siRNF115*) for 24 h, the levels of both *RNF115* and *LC3B* mRNA were detected by qRT–PCR. The sequence of *sicontrol*: UUCUCCGAACGUGUCACGUTT; *siRNF115*: UCUGAAUAGAAUUAAUUAATT. ***$p < 0.001$, ns： no significance.

Figure S8

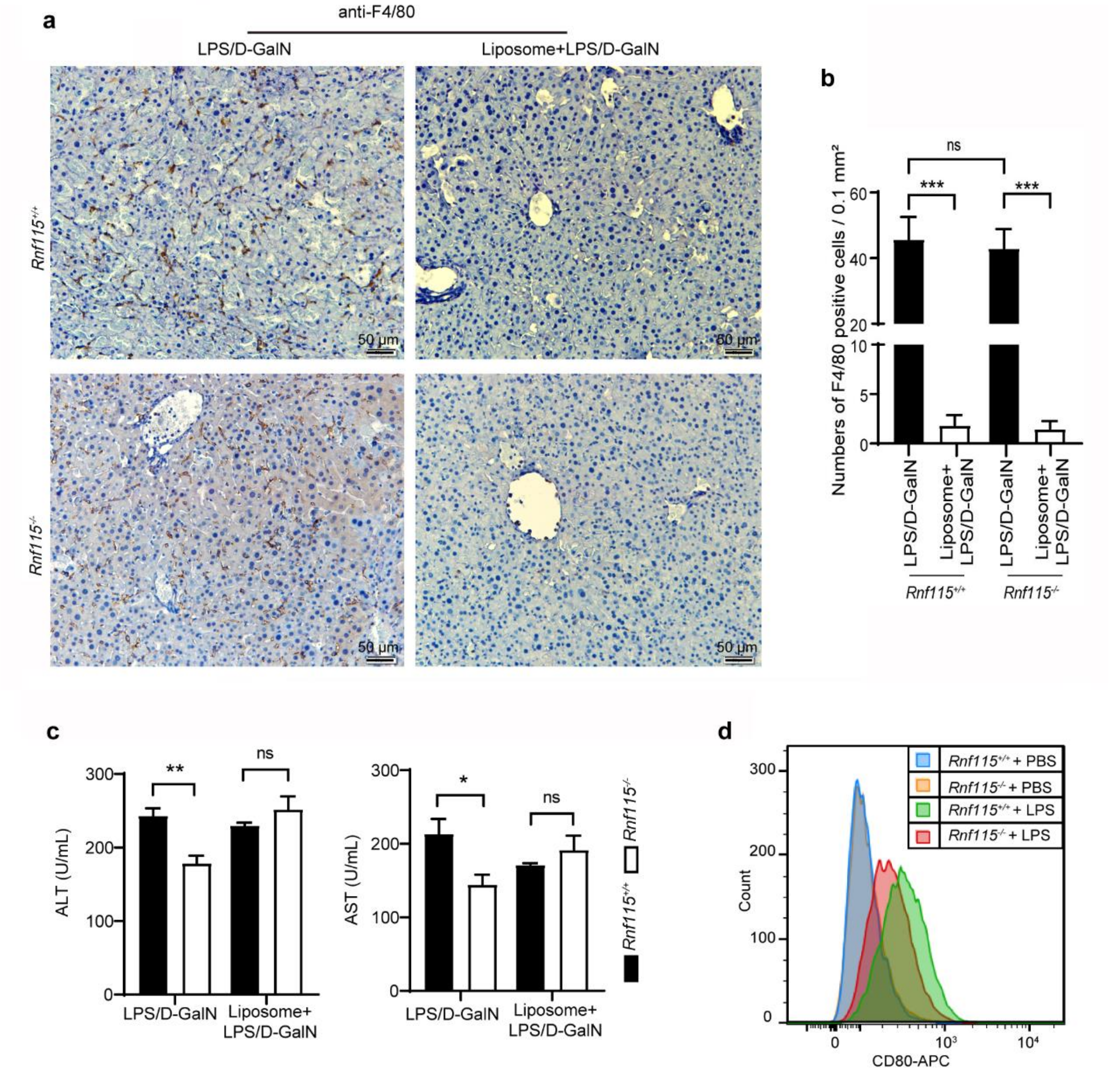

Fig. S8 Depletion of macrophages narrowed the difference in liver injury between *Rnf115*$^{+/+}$ and *Rnf115*$^{-/-}$ mice. (a) *Rnf115*$^{+/+}$ and *Rnf115*$^{-/-}$ mice were treated with Liposome for 48 h before LPS/D-GalN treatment for 5 h. F4/80^{+} cells were detected by immunohistochemical analysis. (b) Numbers of F4/80+ cells in 0.1 mm^2 area were quantificated and statistically analyzed (n = 12). (c) Serum ALT and AST levels from different mice. (d) Flow cytometry analysis of Cd80 expression from *Rnf115*$^{+/+}$ and *Rnf115*$^{-/-}$ BMDMs with or without 100 ng/ml of LPS for 24 h. *$p < 0.05$, **$p < 0.01$, ***p<0.001, ns: no significance.

Figure S9

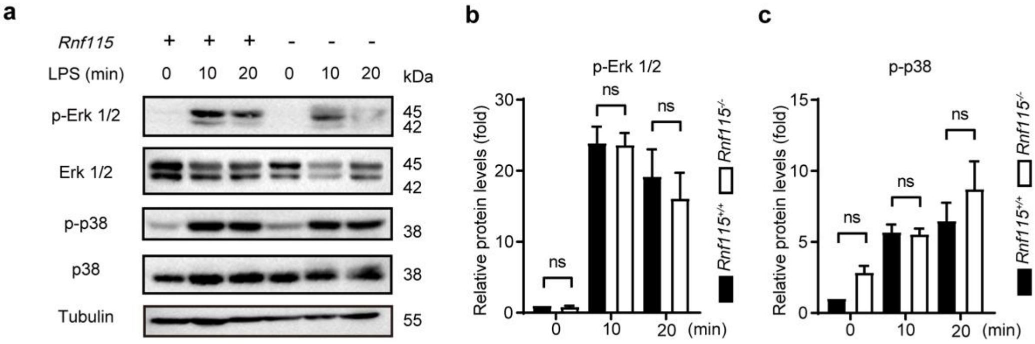

Fig. S9 *Rnf115* knockout failed to affect the activities of Erk 1/2 and p38 Mapk. (a) *Rnf115*$^{+/+}$and *Rnf115*$^{-/-}$BMDMs were treated with or without 100 ng/ml of LPS for the indicated time. Immunoblotting analysis of the expression of indicated proteins. (b and c) Quantification of amounts of indicated protein relative to Tubulin in cells. The average value of *Rnf115*$^{+/+}$mice without LPS was normalized to 1. Data are means ± SD of results from 3 experiments. ns: no significance.

Figure S10

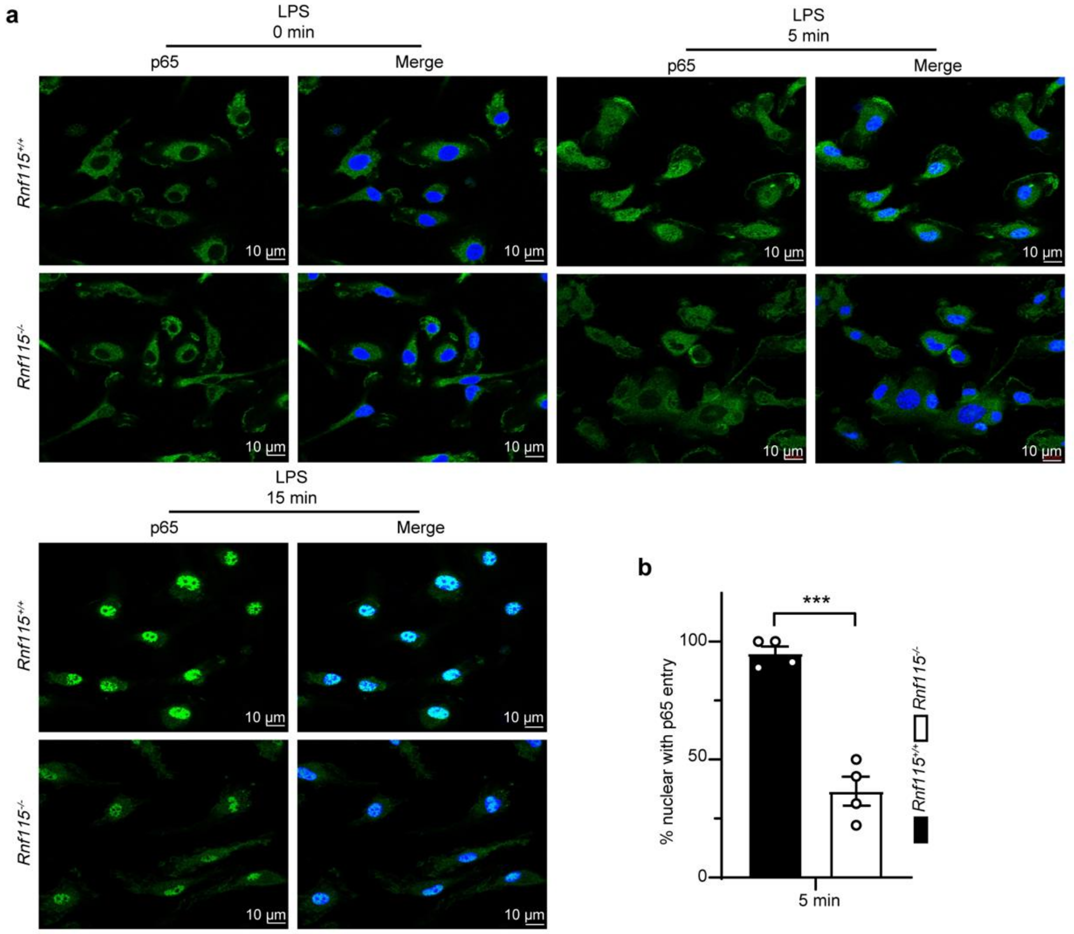

Fig. S10 ***Rnf115*** **knockout declined the fluorescence intensity and nuclear distribution of NF-κB p65 subunit in LPS-stimulated BMDMs**. (a-c) *Rnf115*$^{+/+}$ and *Rnf115*$^{-/-}$ BMDMs were treated with or without LPS (100 ng/mL) for the indicated time, stained by anti- NF-κB p65 antibody and observed by fluorescence microscopy. Cell nuclei were stained with Hoechst 33342. (d) BMDMs were treated as (b). The percentage of nuclear distribution of NF-κB p65 was detected. Data are means ± SD of results from 3 experiments. ***$p < 0.001$.
