# Supplementary material for: RNF115/BCA2 deficiency alleviated acute liver injury in mice by promoting autophagy and inhibiting inflammatory response
Source: Cell Death Dis. 2023 Dec 21;14(12):855. doi: 10.1038/s41419-023-06379-7 (PMC10739886; doi:10.1038/s41419-023-06379-7)
Supplement: Supplementary file 2 — Supplementary Table 1 [file 41419_2023_6379_MOESM2_ESM.docx]

| **Table.1 List of antibodies and reagents used in this manuscript** | | |
| --- | --- | --- |
| Name | Art. No of the Products | Company |
| Anti-LC3B | L7543 | Sigma Aldrich |
| Anti-RNF115 | HPA019130 |  |
| Anti-NF-κB/p65 | 8242 | Cell Signaling technology |
| Anti-Phospho- NF-κB/p65 (Ser536) | 3033 |  |
| Anti-p38/MAPK | 8690 |  |
| Anti-Phospho-p38/MAPK (Thr180/Tyr182) | 4511 |  |
| Anti-p44/42 MAPK (ERK1/2) | 4695 |  |
| Anti-Phospho-p44/42 MAPK (ERK1/2) (Thr202/Tyr204) | 4370s |  |
| Anti-JNK2 | 9258s |  |
| Anti-Phospho-JNK(Thr183/Tyr185) | 9255s |  |
| Anti-iNOS | 13120 |  |
| Anti-β-Tubulin | KM9003 | Tianjin Sungene Biotech |
| Anti-GFP | KM8009L |  |
| Anti-GST | KM8005 |  |
| Anti- FLAG | KM8002 |  |
| Anti-β-actin | TA-09 | OriGene |
| Anti- His | BSM-33004M | Bioss Inc. |
| Anti-GAPDH | BSM-0978M |  |
| Goat Anti-Rabbit IgG Antibody (H+L), HRP | bs-40295G |  |
| Goat Anti-Mouse IgG Antibody (H+L)，HRP | bs-40296G |  |
| Anti- SQSTM1/p62 | PM045 | MBL |
| Anti-Beclin1 | PD017 |  |
| Anti-T7 | 69522 | Millipore |
| APC anti-mouse CD80 Antibody | 104713 | BioLegend |
| APC anti-mouse Ly-6G Antibody | 127613 |  |
| FITC anti-mouse CD45 Antibody | 103107 |  |
| PE anti-mouse F4/80 Antibody | 123109 |  |
| PE/Cyanine7 anti-mouse Ly-6C Antibody | 128017 |  |
| PerCP anti-mouse/human CD11b Antibody | 101229 |  |
| PE-Cy™7 Rat Anti-Mouse CD19 | 552854 | Becton & Dickinson |
| Anti -F4/80 Rabbit pAb | GB113373 | Servicebio |
| Anti -Ly6g Rabbit pAb | GB11229 |  |
| EBSS (Earles’s Blanced salts solution) | 24010043 | Invitrogen |
| Bafilomycin A1 (Baf.A1) | B1793 | Sigma Aldrich |
| MG132 | C2211 |  |
| LPS (E.coli O111:B4)(animal) | L2630 |  |
| Hoechst 33342 | 14533 |  |
| D-(+)-Galactosamine hydrochloride | G1639 |  |
| Clodronate Liposomes | CP-005 | Liposoma |
| 3-MA | SR2721 | HARVEYBIO |
| Cycloheximide | 239763 | Calbiochem |
| LPS (E.coli K12)(cell) | LPS-EK | InvivoGen |
