# Supplementary material for: RNF115/BCA2 deficiency alleviated acute liver injury in mice by promoting autophagy and inhibiting inflammatory response
Source: Cell Death Dis. 2023 Dec 21;14(12):855. doi: 10.1038/s41419-023-06379-7 (PMC10739886; doi:10.1038/s41419-023-06379-7)
Supplement: Supplementary file 3 — Supplementary Table 2 [file 41419_2023_6379_MOESM3_ESM.docx]

## Supplementary Table 2. The qRT-PCR Primer sequences used in this study

| Name | | Sequence |
| --- | --- | --- |
| *Rnf115* | Forward Primer | 5’- CTTCTGCCACTTTTGTAAGGGC -3’ |
|  | Reverse Primer | 5’- ACTGGAATCATCTGTCACTTCCT -3’ |
| *Actb* | Forward Primer | 5’- GGCTGTATTCCCCTCCATCG - 3’ |
|  | Reverse Primer | 5’- CCAGTTGGTAACAATGCCATGT -3’ |
| *Tnf* | Forward Primer | 5’- CCCTCACACTCAGATCATCTTCT -3’ |
|  | Reverse Primer | 5’- GCTACGACGTGGGCTACAG -3’ |
| *Il-6* | Forward Primer | 5’- CTGCAAGAGACTTCCATCCAG -3’ |
|  | Reverse Primer | 5’- AGTGGTATAGACAGGTCTGTTGG -3’ |
| *Cd80* | Forward Primer | 5’- GCAGGATACACCACTCCTCAA -3’ |
|  | Reverse Primer | 5’- AAAGACGAATCAGCAGCACAA -3’ |
| *Nos2* | Forward Primer | 5’- GTTCTCAGCCCAACAATACAAGA -3’ |
|  | Reverse Primer | 5’- GTGGACGGGTCGATGTCAC -3’ |
| *Mcp1* | Forward Primer | 5’- TTAAAAACCTGGATCGGAACCAA -3’ |
|  | Reverse Primer | 5’- GCATTAGCTTCAGATTTACGGGT -3’ |
| *Il-1β* | Forward Primer | 5’- GCAACTGTTCCTGAACTCAACT -3’ |
|  | Reverse Primer | 5’- ATCTTTTGGGGTCCGTCAACT -3’ |
| *Ifnβ1* | Forward Primer | 5’- CAGCTCCAAGAAAGGACGAAC -3’ |
|  | Reverse Primer | 5’- GGCAGTGTAACTCTTCTGCAT -3’ |
| *LC3B* | Forward Primer | 5’- AAGGCGCTTACAGCTCAATG -3’ |
|  | Reverse Primer | 5’- CTGGGAGGCATAGACCATGT -3’ |
| *ACTB* | Forward Primer | 5’- CATGTACGTTGCTATCCAGGC -3’ |
|  | Reverse Primer | 5’- CTCCTTAATGTCACGCACGAT-3’ |
