# Supplementary material for: RNF115/BCA2 deficiency alleviated acute liver injury in mice by promoting autophagy and inhibiting inflammatory response
Source: Cell Death Dis. 2023 Dec 21;14(12):855. doi: 10.1038/s41419-023-06379-7 (PMC10739886; doi:10.1038/s41419-023-06379-7)

Original data of Western blot

Figure 3d

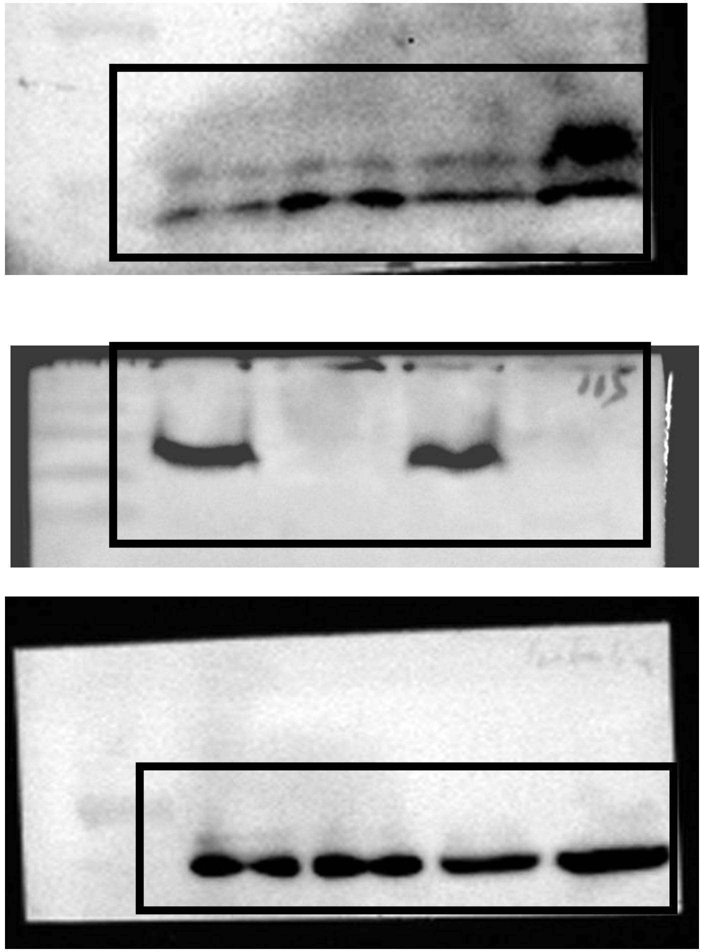

Figure 3e

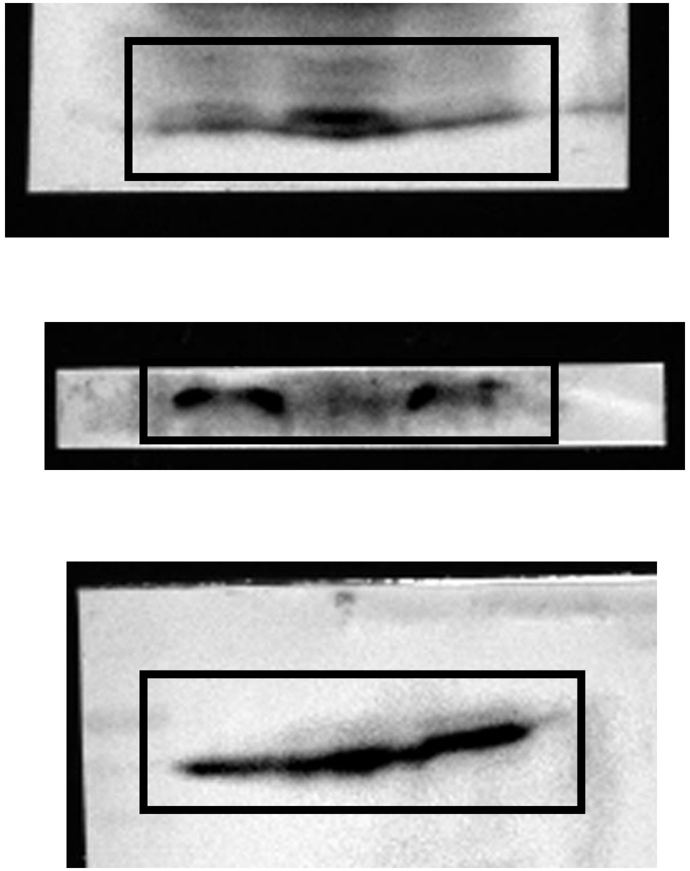

Figure 4a

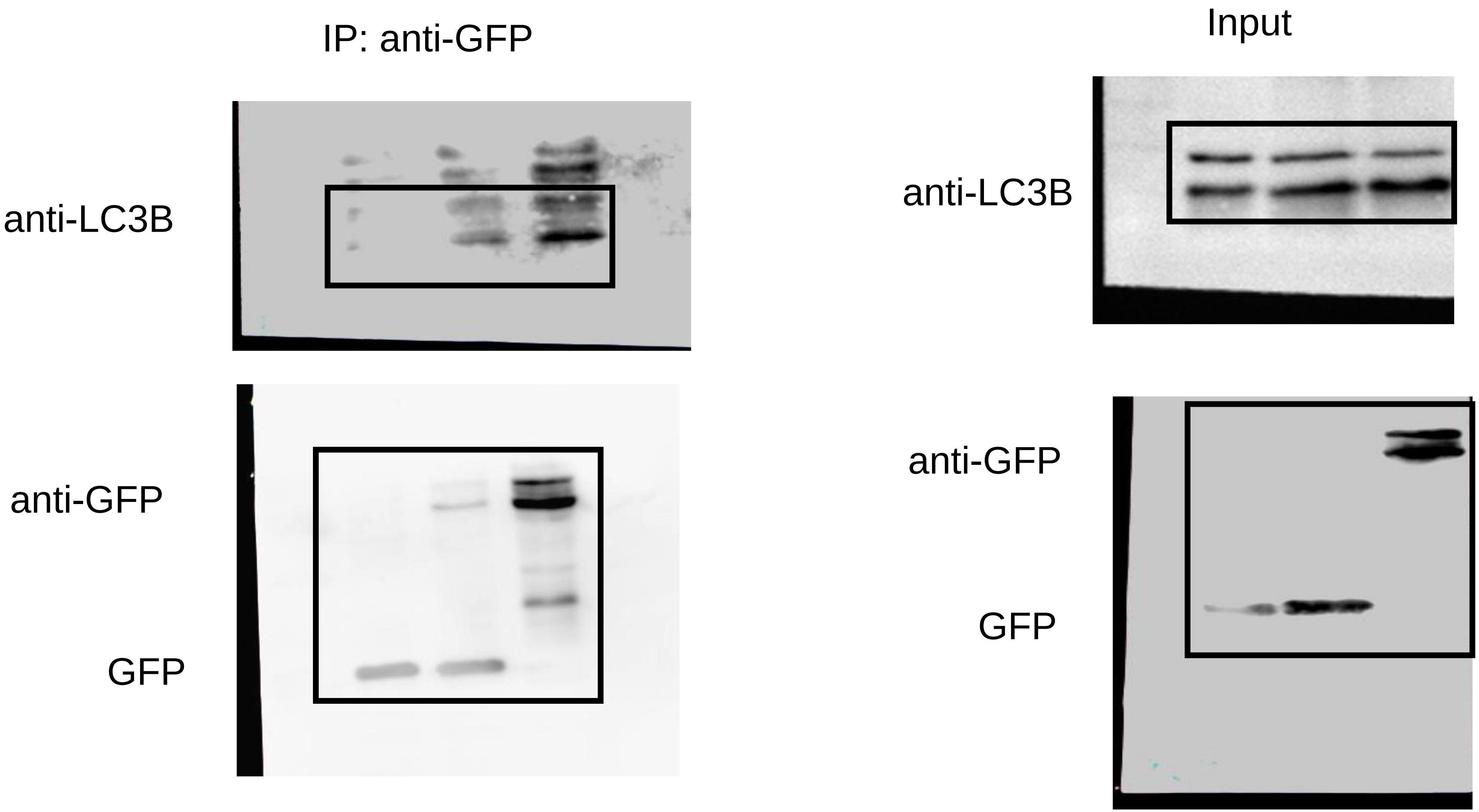
IP: anti-GFP
Input
anti-LC3B
anti-LC3B
anti-GFP
anti-GFP
GFP
GFP

Figure 4b

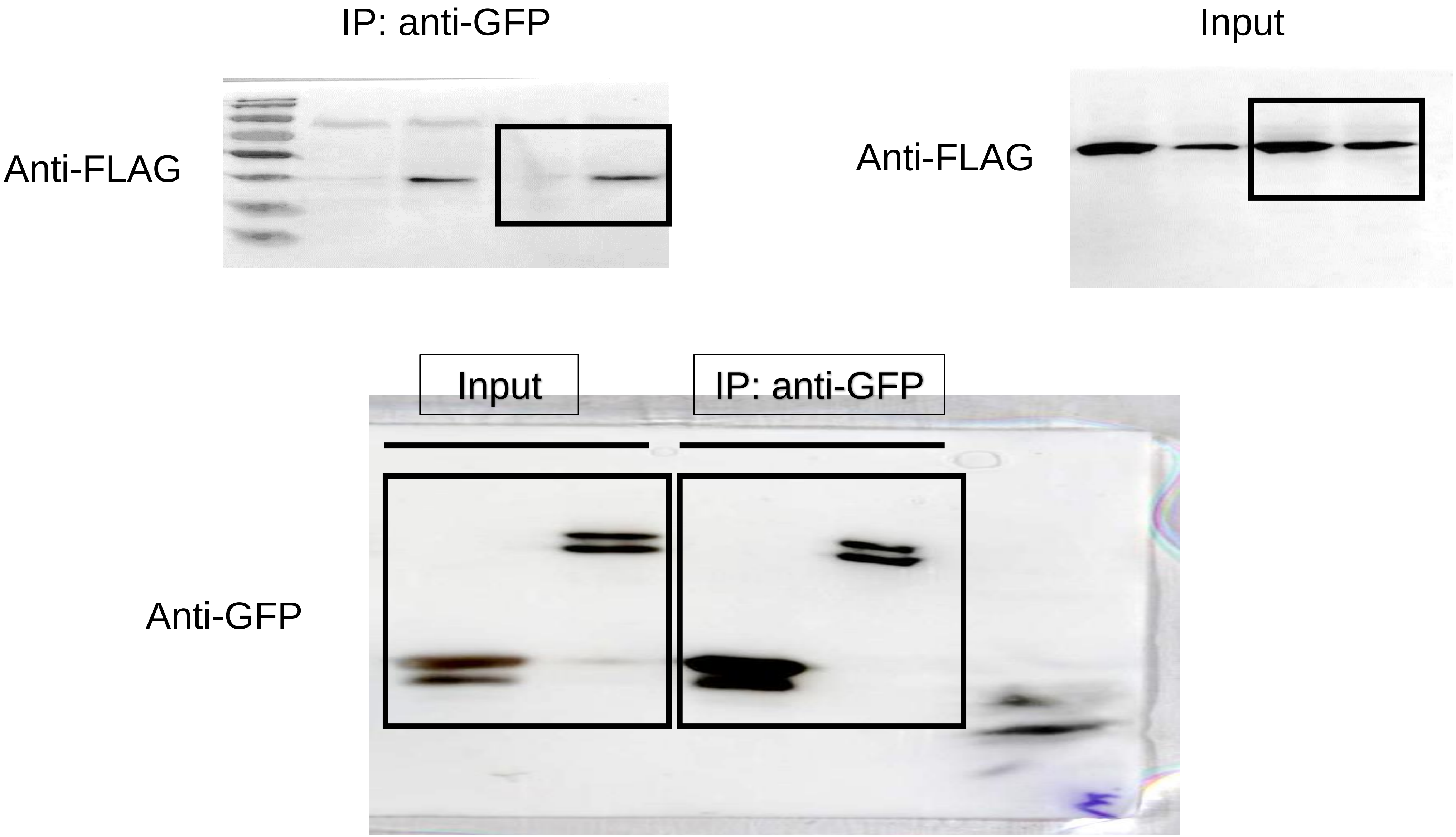
IP: anti-GFP
Input
Anti-FLAG
Anti-FLAG
Input
IP: anti-GFP
Anti-GFP

Figure 4c

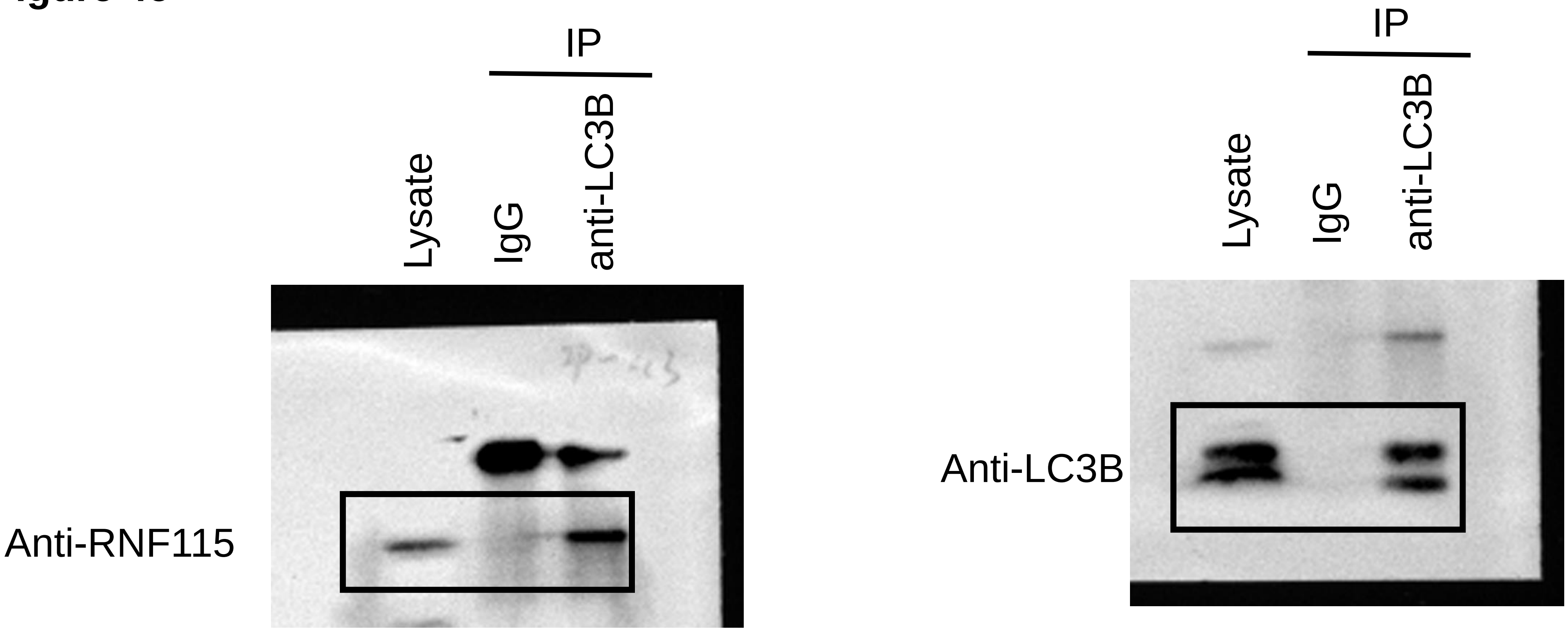
IP
Lysate
IgG
anti-LC3B
Anti-RNF115
IP
Lysate
IgG
anti-LC3B
Anti-LC3B

Figure 4d

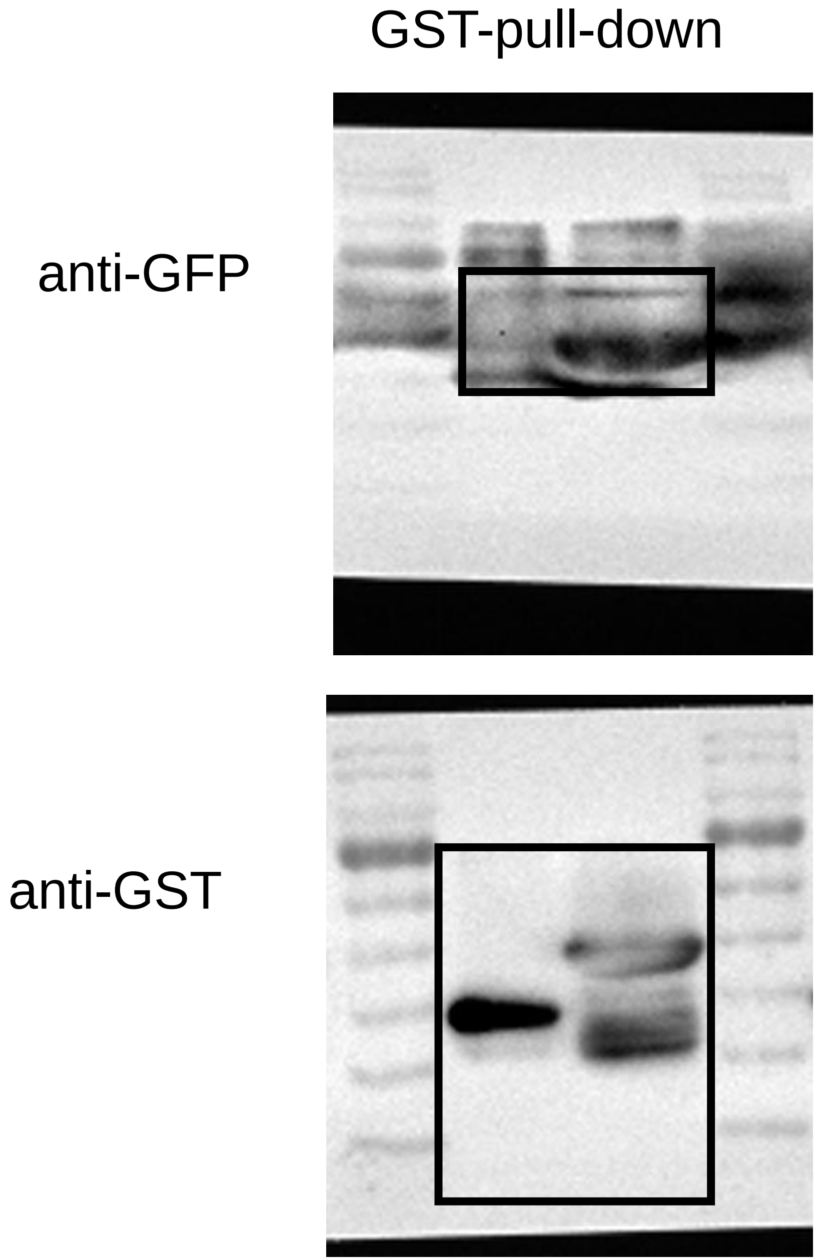

Figure 4e

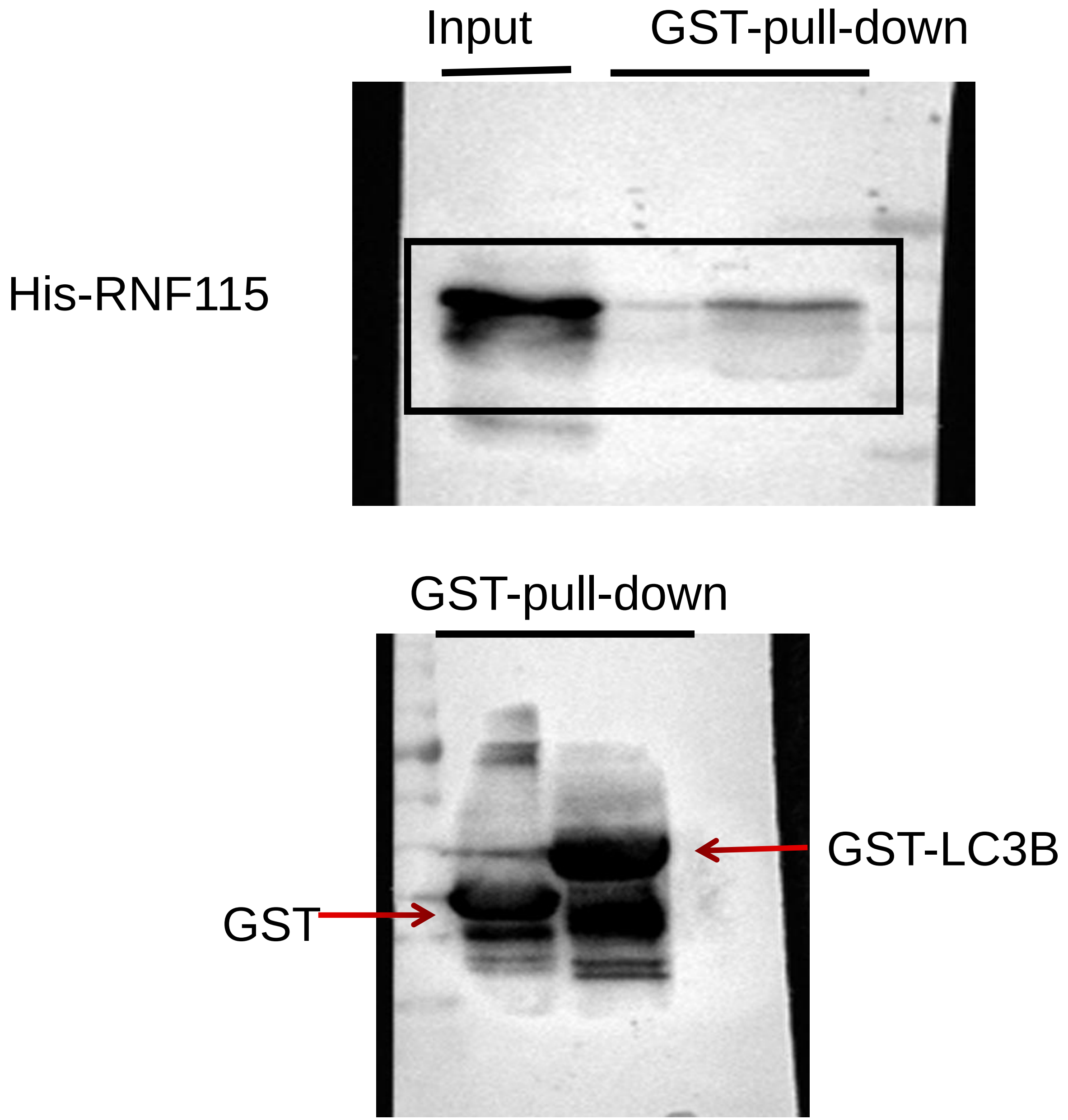

Figure 4g

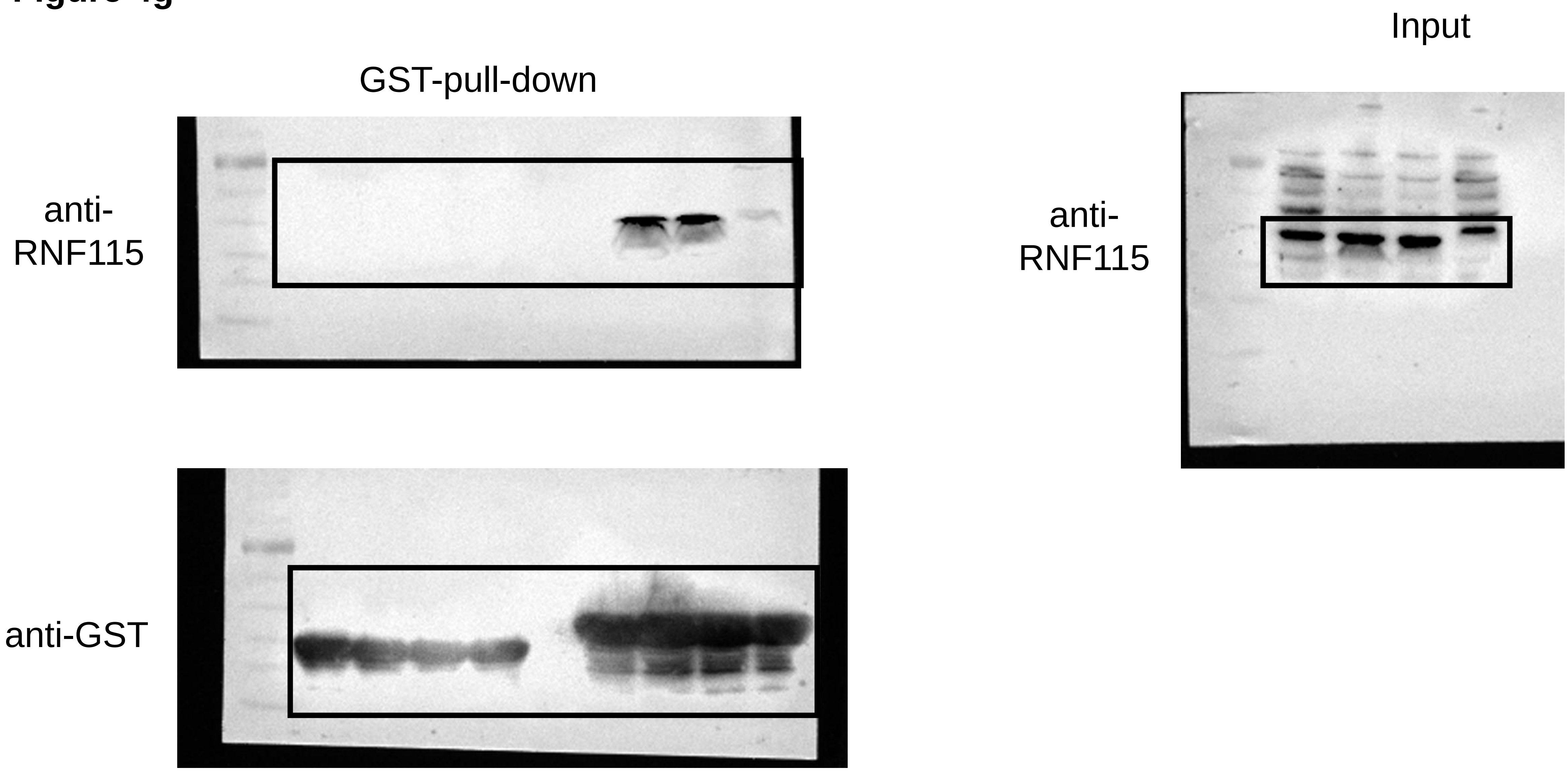

Figure 4h

GST-pull-down

GFP-RNF115

anti-GST

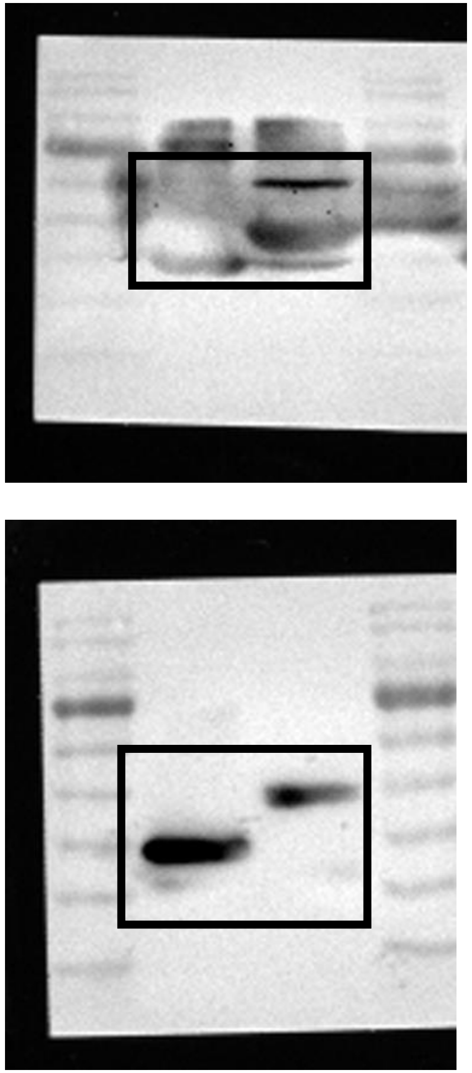

Figure 4i

GST-pull-down

anti-GFP

Anti-GST

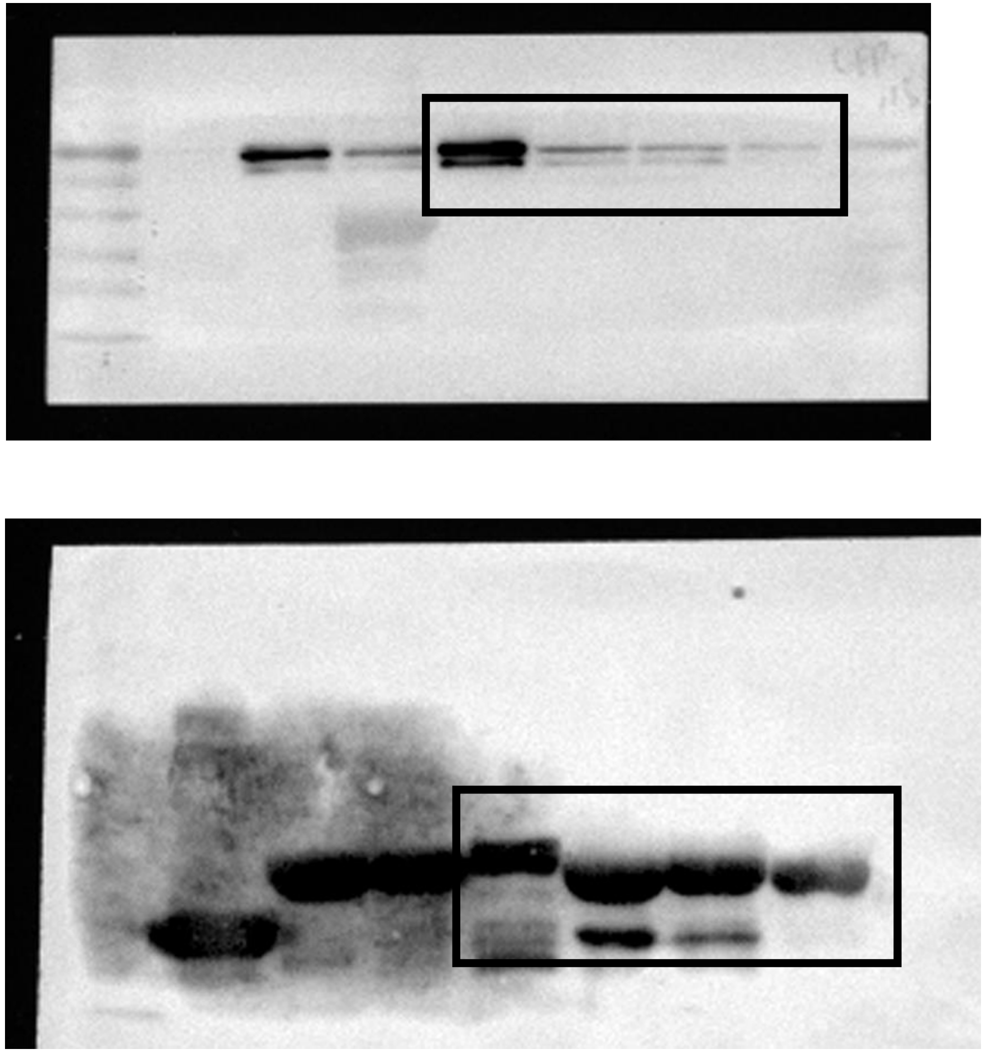

Figure 5a

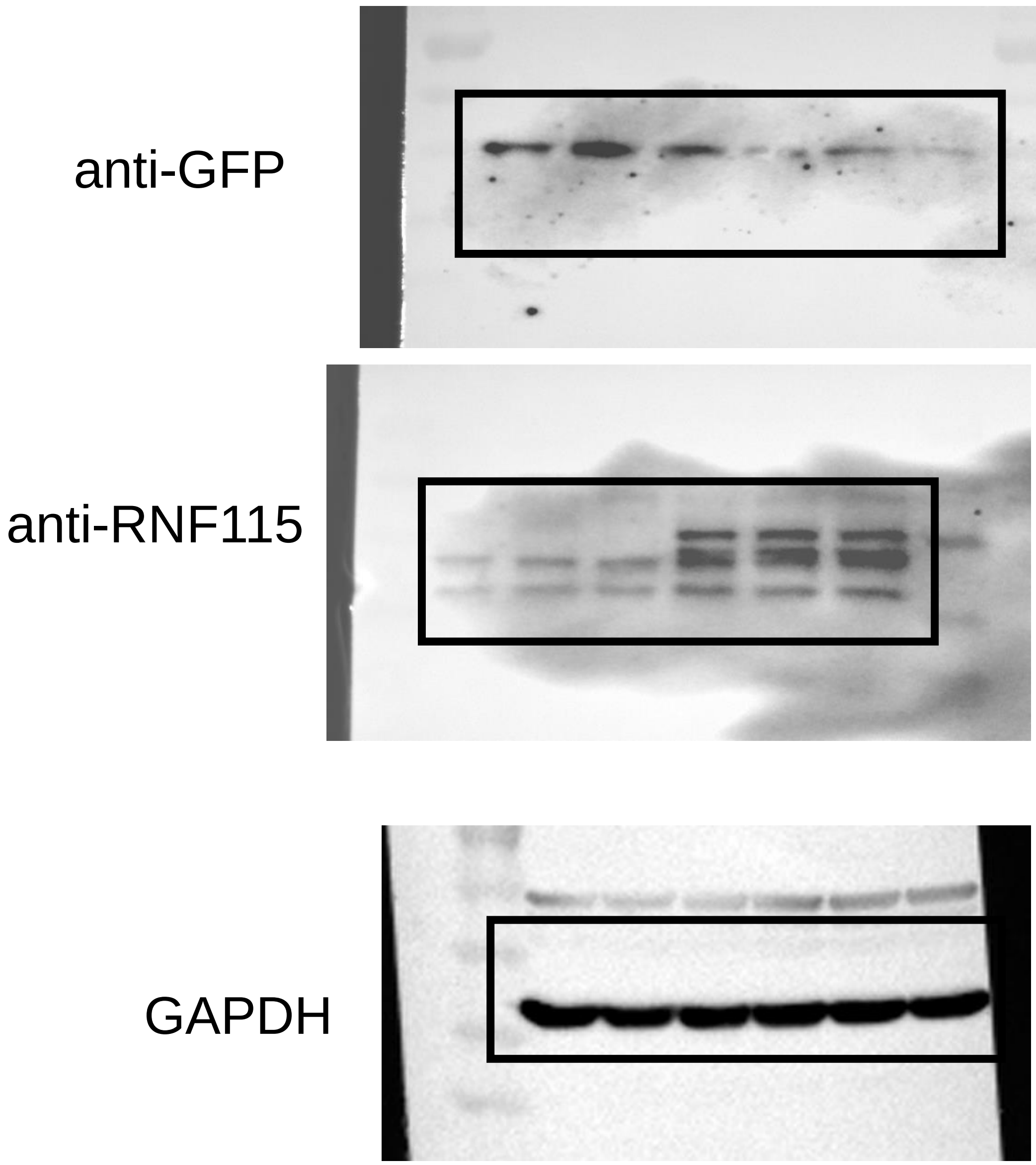

Figure 5b

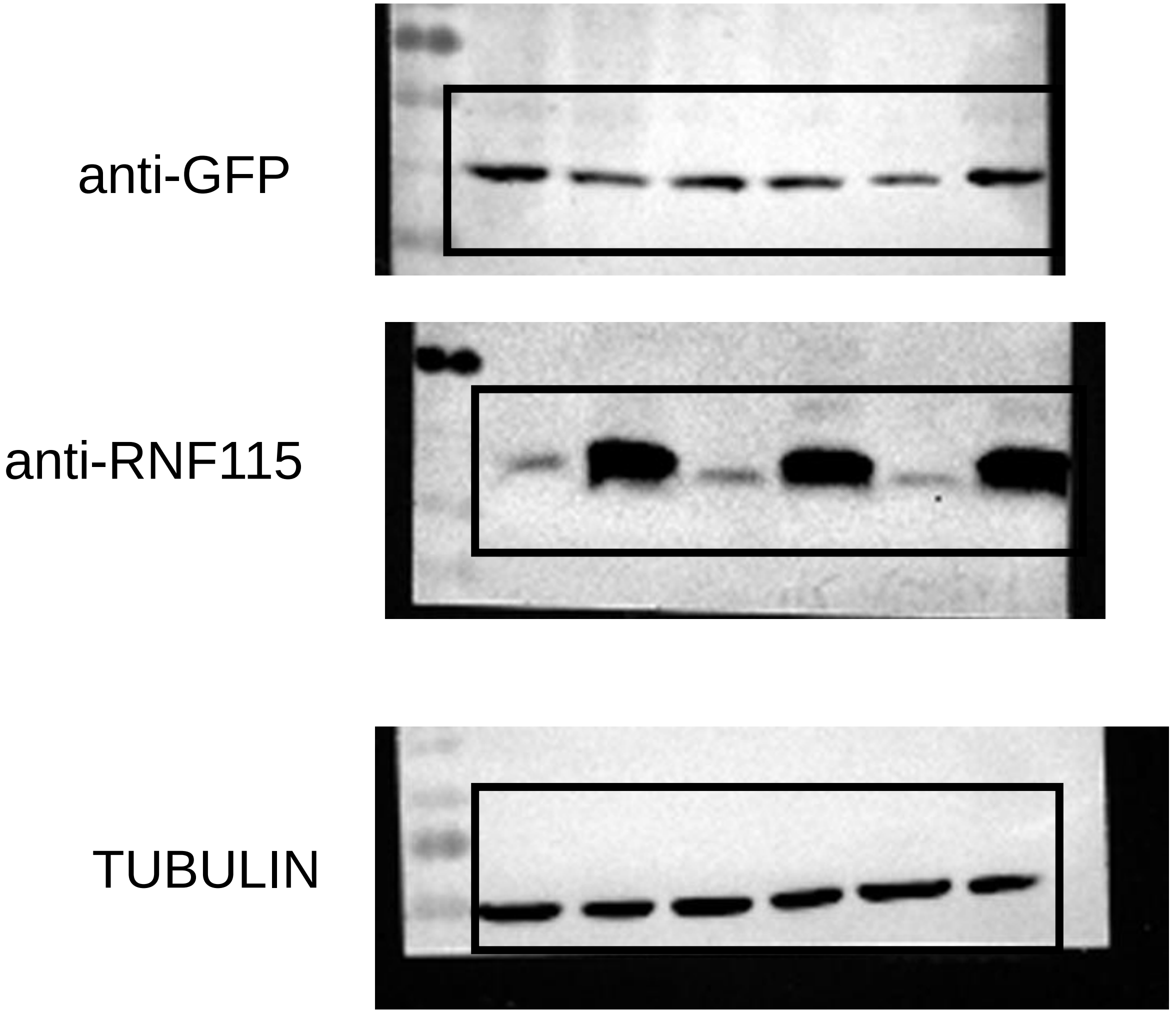

Figure 5c

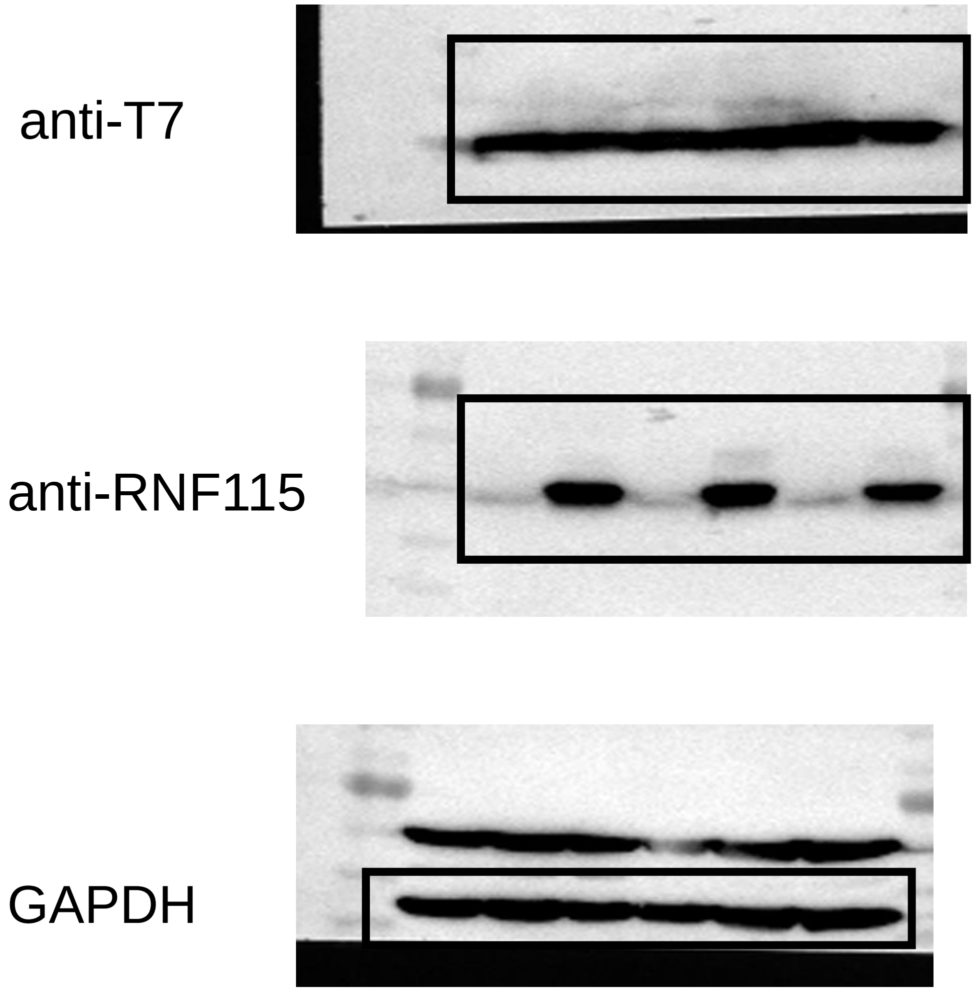

Figure 5d

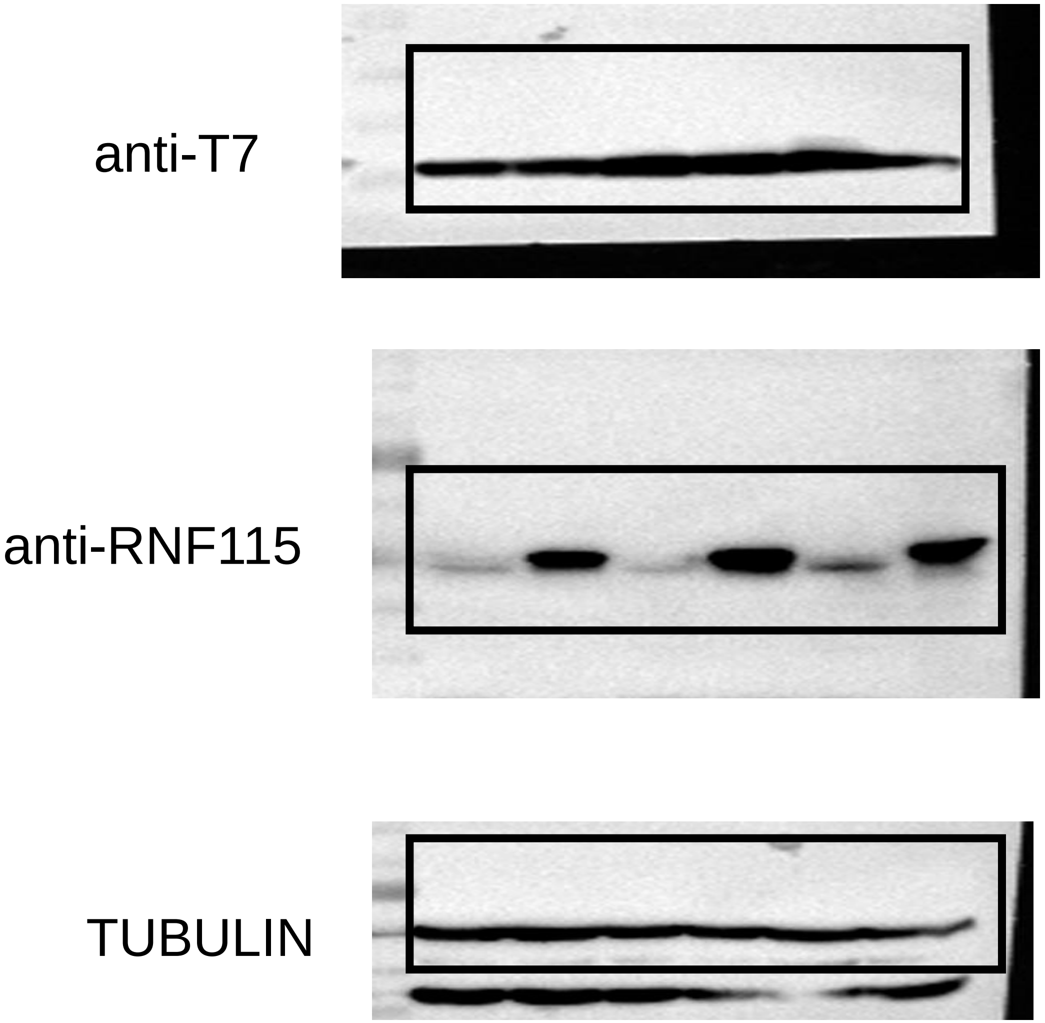

Figure 6c

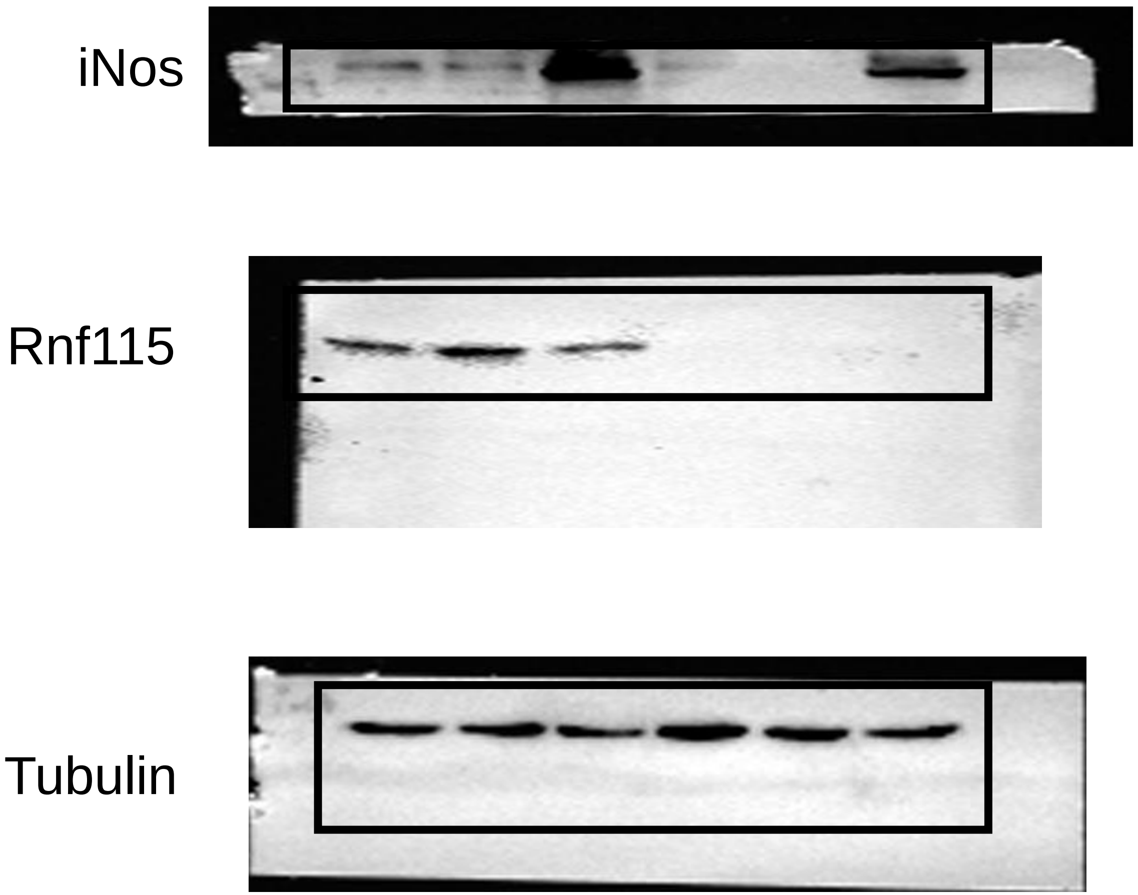

Figure 6e

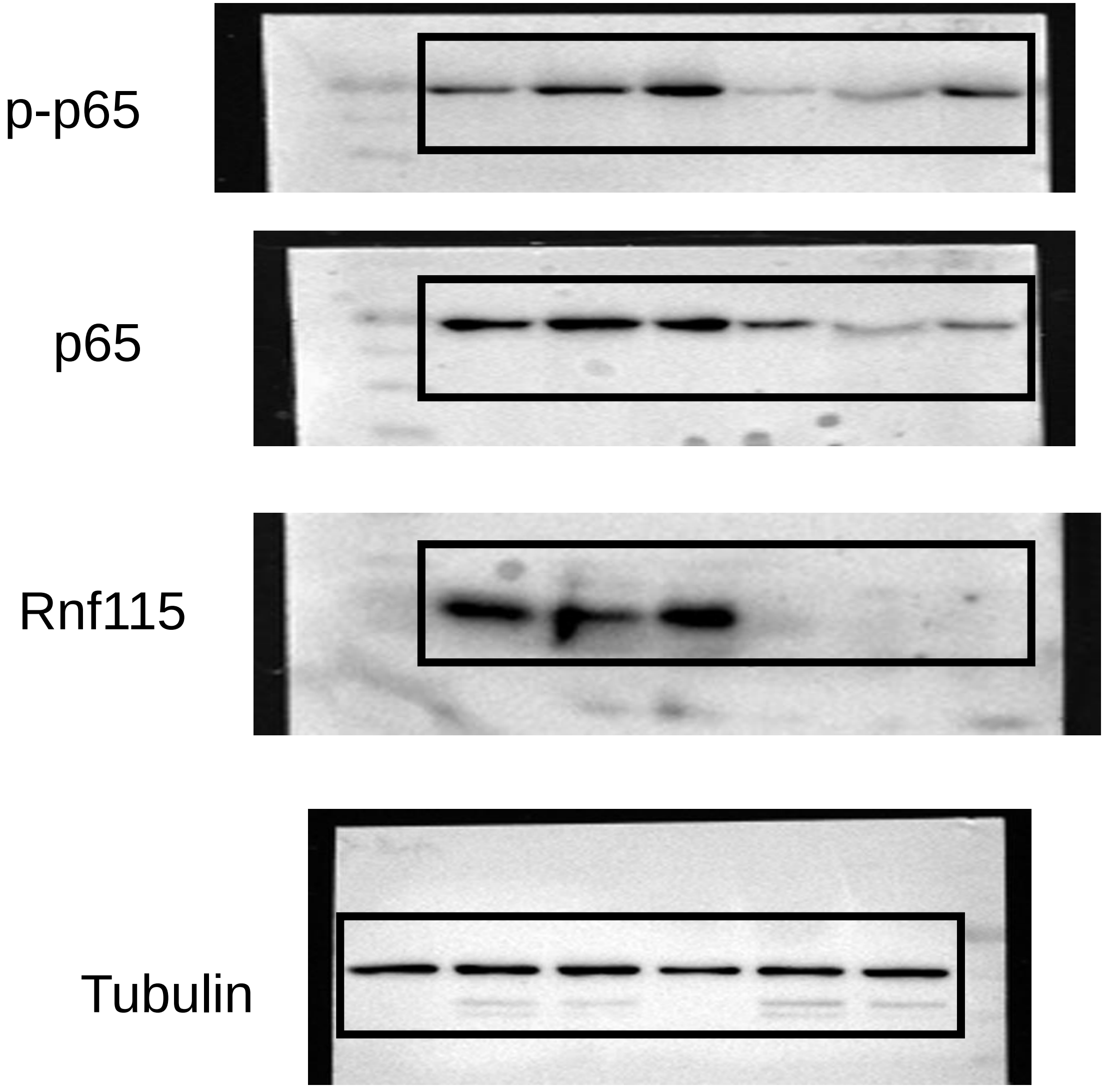

Figure 6g

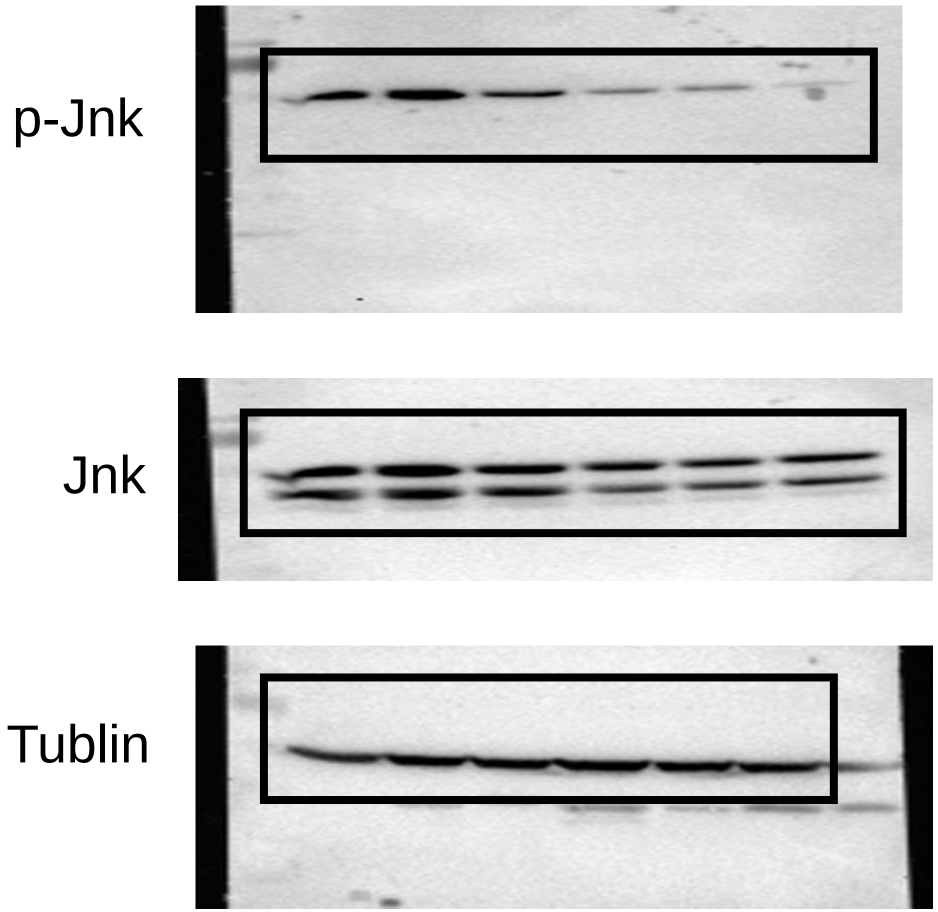

Figure 6i

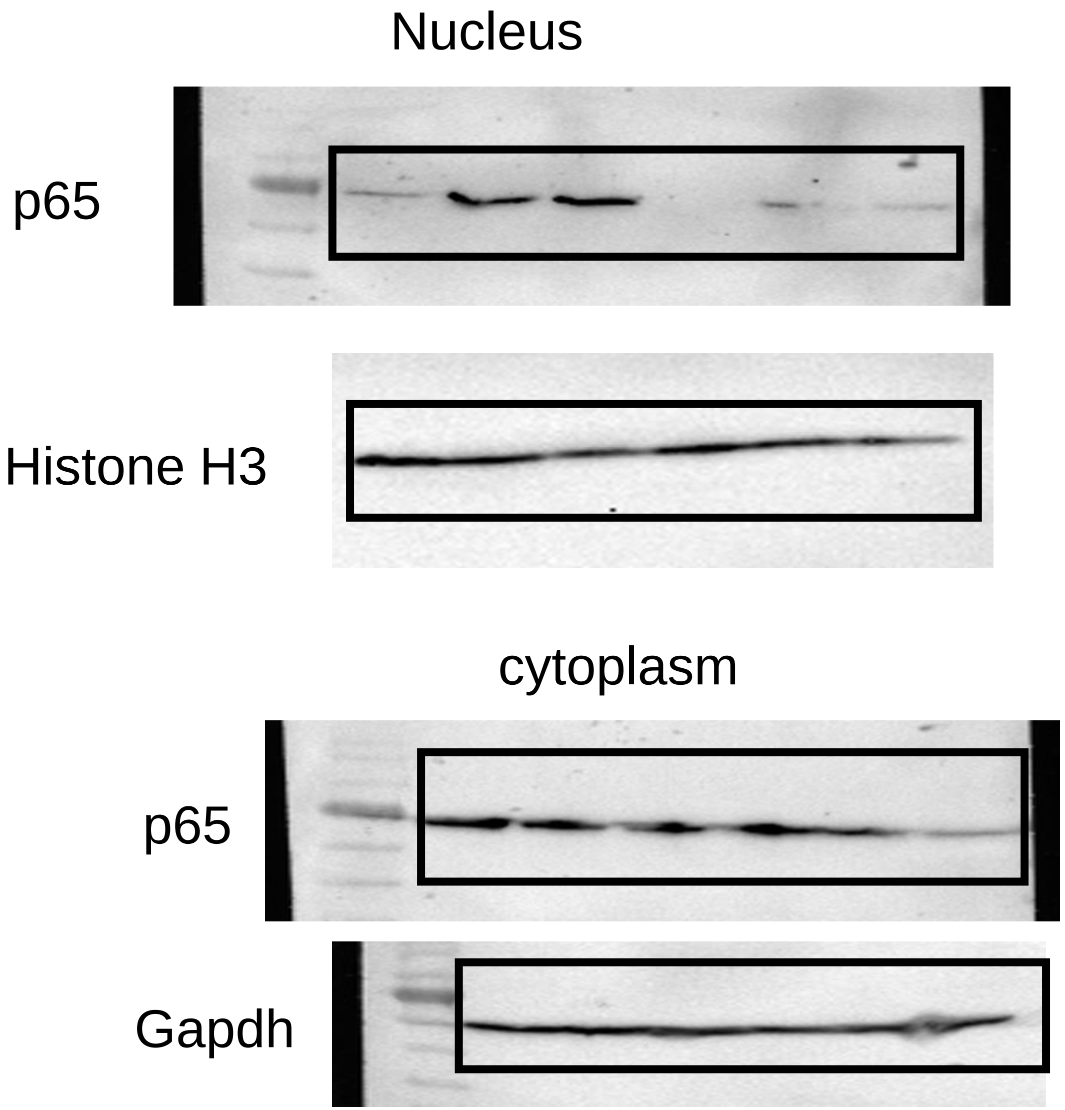

Figure S1

Rnf115

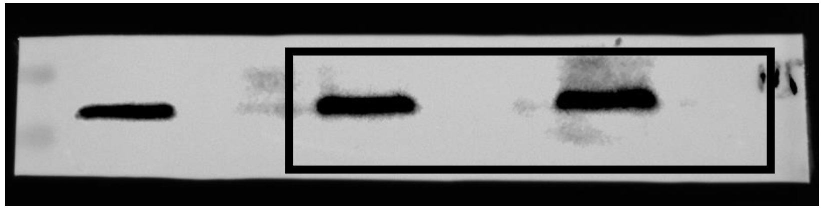

Actb

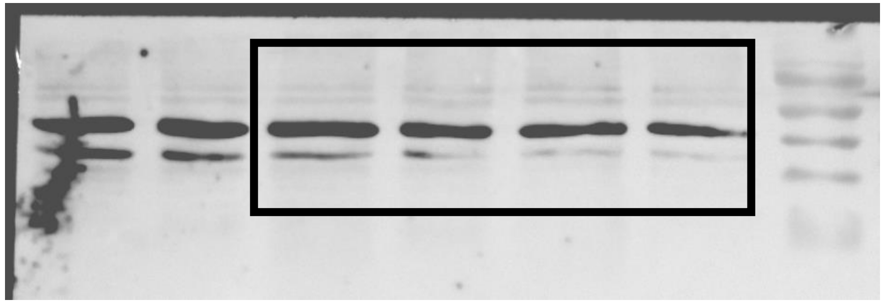

Figure S6

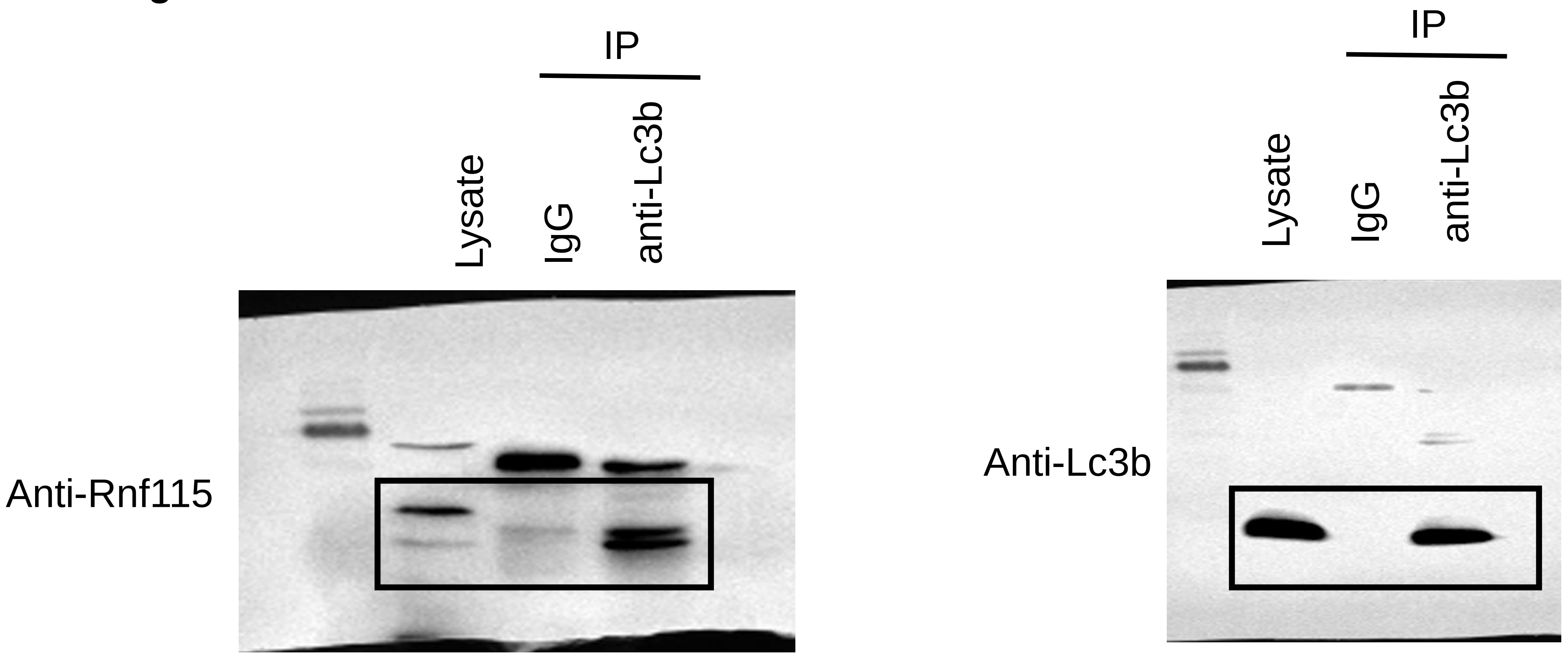
IP
Lysate
IgG
anti-Lc3b
Anti-Rnf115
IP
Lysate
IgG
anti-Lc3b
Anti-Lc3b

Figure S7a

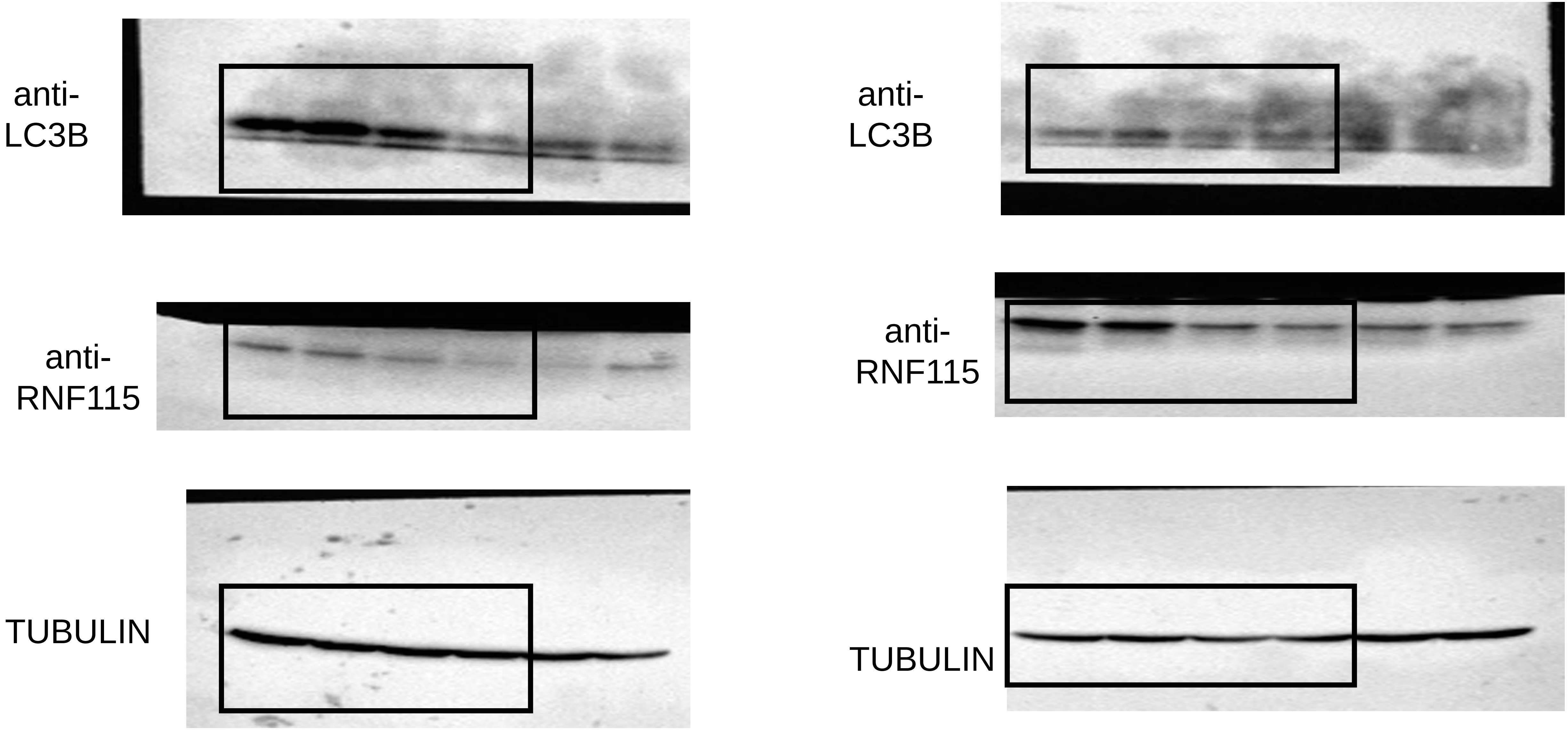
anti-
LC3B
anti-
RNF115
TUBULIN
anti-
LC3B
anti-
RNF115
TUBULIN

Figure S9a

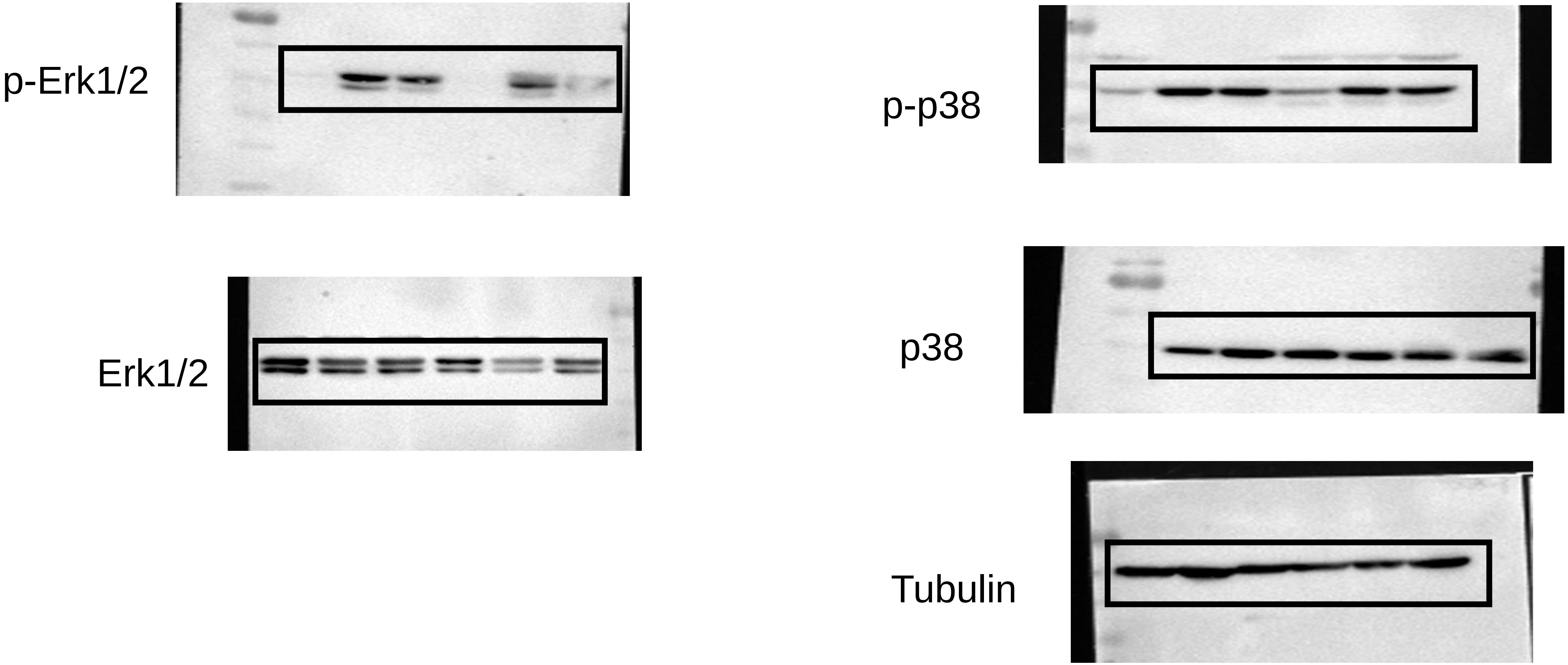

Supplement: Supplementary file 4 — Original Data File [file 41419_2023_6379_MOESM4_ESM.pdf]
